# Non-canonical Activation of the DNA Sensing Adaptor STING by ATM and IFI16 Mediates NF-κB Signaling after Nuclear DNA Damage

**DOI:** 10.1016/j.molcel.2018.07.034

**Published:** 2018-09-06

**Authors:** Gillian Dunphy, Sinéad M. Flannery, Jessica F. Almine, Dympna J. Connolly, Christina Paulus, Kasper L. Jønsson, Martin R. Jakobsen, Michael M. Nevels, Andrew G. Bowie, Leonie Unterholzner

**Affiliations:** 1 Division of Biomedical and Life Sciences, Faculty of Health and Medicine, Lancaster University, Lancaster LA1 4YQ, UK; 2 School of Biochemistry and Immunology, Trinity Biomedical Sciences Institute, Trinity College Dublin, Dublin 2, Ireland; 3 Biomedical Sciences Research Complex, University of St Andrews, St Andrews KY16 9ST, UK; 4 Department of Biomedicine, Aarhus University, Wilhelm Meyers Allé 4, 8000 Aarhus C, Denmark

**Keywords:** innate immunity, DNA damage, etoposide, interferon, IFI16, STING, p53, TRAF6, ubiquitin

## Abstract

DNA damage can be sensed as a danger-associated molecular pattern by the innate immune system. Here we find that keratinocytes and other human cells mount an innate immune response within hours of etoposide-induced DNA damage, which involves the DNA sensing adaptor STING but is independent of the cytosolic DNA receptor cGAS. This non-canonical activation of STING is mediated by the DNA binding protein IFI16, together with the DNA damage response factors ATM and PARP-1, resulting in the assembly of an alternative STING signaling complex that includes the tumor suppressor p53 and the E3 ubiquitin ligase TRAF6. TRAF6 catalyzes the formation of K63-linked ubiquitin chains on STING, leading to the activation of the transcription factor NF-κB and the induction of an alternative STING-dependent gene expression program. We propose that STING acts as a signaling hub that coordinates a transcriptional response depending on its mode of activation.

## Highlights

• Etoposide-induced nuclear DNA damage causes an innate immune response
• ATM and IFI16 can activate STING in a cGAS-independent manner
• Non-canonical STING signaling predominantly activates NF-κB, rather than IRF3
• This involves the TRAF6-mediated assembly of K63-linked ubiquitin chains on STING

## Graphical Abstract

**Figure:**
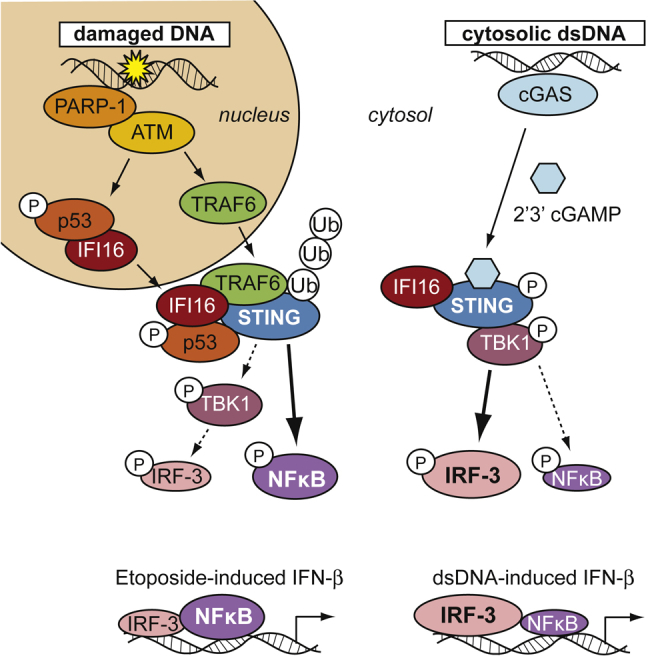

## Teaser

DNA damage can activate an innate immune response. Dunphy et al. show that the detection of etoposide-induced damage by ATM causes the non-canonical activation of the DNA sensing adaptor STING. This results in the assembly of an alternative STING signaling complex and the induction of an NF-κB-dependent transcriptional program.

## Introduction

The innate immune system provides a rapid initial defense program against invading pathogens that relies on the recognition of pathogen-associated molecular patterns (PAMPs) to shape local immune responses. Innate immune activation can also be observed in the absence of infection, following the detection of danger-associated molecular patterns (DAMPs) after injury or during sterile inflammation. PAMPs and DAMPs are detected by pattern recognition receptors, which then induce the production of interferons, cytokines, and chemokines. Intracellular DNA receptors recognize double-stranded (ds) DNA as a PAMP during infection with DNA viruses and other intracellular pathogens but can also detect self-DNA as a DAMP under some circumstances, for instance, when damaged DNA has leaked into the cytosol (reviewed by Dhanwani et al., 2018). The key DNA sensor in the cytosol is cyclic guanosine monophosphate (GMP)-AMP synthase (cGAS), which catalyzes the synthesis of the second messenger cyclic GMP-AMP (cGAMP) (reviewed by Chen et al., 2016). cGAMP then binds to the DNA sensing adaptor STING at the endoplasmic reticulum (ER), causing a conformational change associated with the activation of STING dimers (Cai et al., 2014). In addition to the conformational change caused by cGAMP binding, STING function is regulated by phosphorylation, palmitoylation, sumoylation, and modification with K63-, K48-, K27-, and K11-linked poly-ubiquitin chains (Chiang and Gack, 2017, Hu et al., 2016, Mukai et al., 2016). Following its activation, STING translocates from the ER to signaling compartments, where STING associates with the kinase TBK1, which mediates the activation of the transcription factor interferon regulatory factor 3 (IRF3) and, to a lesser extent, nuclear factor κB (NF-κB) (Abe and Barber, 2014, Dobbs et al., 2015, Liu et al., 2015). Both IRF3 and NF-κB are required for the expression of interferon-β (*IFN-β*) mRNA.

cGAS-mediated STING activation is crucial for the detection of DNA from intracellular pathogens. In human monocytes and keratinocytes, STING activation by cGAS requires the cooperation with the DNA binding protein IFI16 (interferon-γ-inducible factor 16), which shuttles between the nucleus and the cytosol but is nuclear at steady state (Almine et al., 2017, Jønsson et al., 2017, Li et al., 2012, Unterholzner et al., 2010). cGAS can also detect DNA damage in a process involving mitotic progression, the formation of micronuclei and leakage of DNA into the cytosol, which occurs several days after recovery from DNA damage (Dou et al., 2017, Glück et al., 2017, Harding et al., 2017, Mackenzie et al., 2017).

dsDNA breaks are also known to induce a more rapid innate immune response that involves the activation of NF-κB (reviewed by Miyamoto, 2011). The transcription factor NF-κB promotes cell survival and can induce the expression of a variety of cytokines and chemokines. DNA damage-induced NF-κB activation can influence tumor progression and clearance of tumor cells by the immune system after radio- or chemotherapy (reviewed by Hellweg, 2015). NF-κB activation occurs within the first hours following the detection of double-strand breaks and involves the DNA damage kinase ataxia telangiectasia mutated (ATM) and the enzyme poly(ADP-ribose) polymerase 1 (PARP-1) (Hinz et al., 2010, Piret et al., 1999, Stilmann et al., 2009). ATM and PARP-1 detect double-strand breaks in the nucleus and signal inside out to activate a cytosolic signaling complex containing the E3 ubiquitin ligase TRAF6, TAK1, TAB2/3, and the NF-κB inhibitor (IκB) kinase complex (IKKα, IKKβ, and NEMO) (Hinz et al., 2010, Stilmann et al., 2009, Wu et al., 2010). ATM-dependent NF-κB activation has been observed in response to ionizing radiation, replication stress, and topoisomerase poisons such as etoposide (reviewed by Miyamoto, 2011).

We find that etoposide treatment induces an NF-κB-dependent innate immune response within hours of treatment, which is particularly potent in human keratinocytes. This response involves ATM and PARP-1, as well as the DNA sensing factors IFI16 and STING, but is independent of cGAS and cGAMP production. Etoposide-induced DNA damage results in a non-canonical mode of STING activation, causing the assembly of an alternative STING-containing signaling complex. This leads to the predominant activation of NF-κB, rather than IRF3, and the induction of an innate immune gene expression profile that differs from that induced by cytosolic DNA sensing. We propose that alternative modes of STING activation shape the innate immune response, depending on the type of threat.

## Results

### Etoposide-Induced DNA Damage Causes an Acute Cell-Intrinsic Innate Immune Response in Human Cells

**Figure 1:**
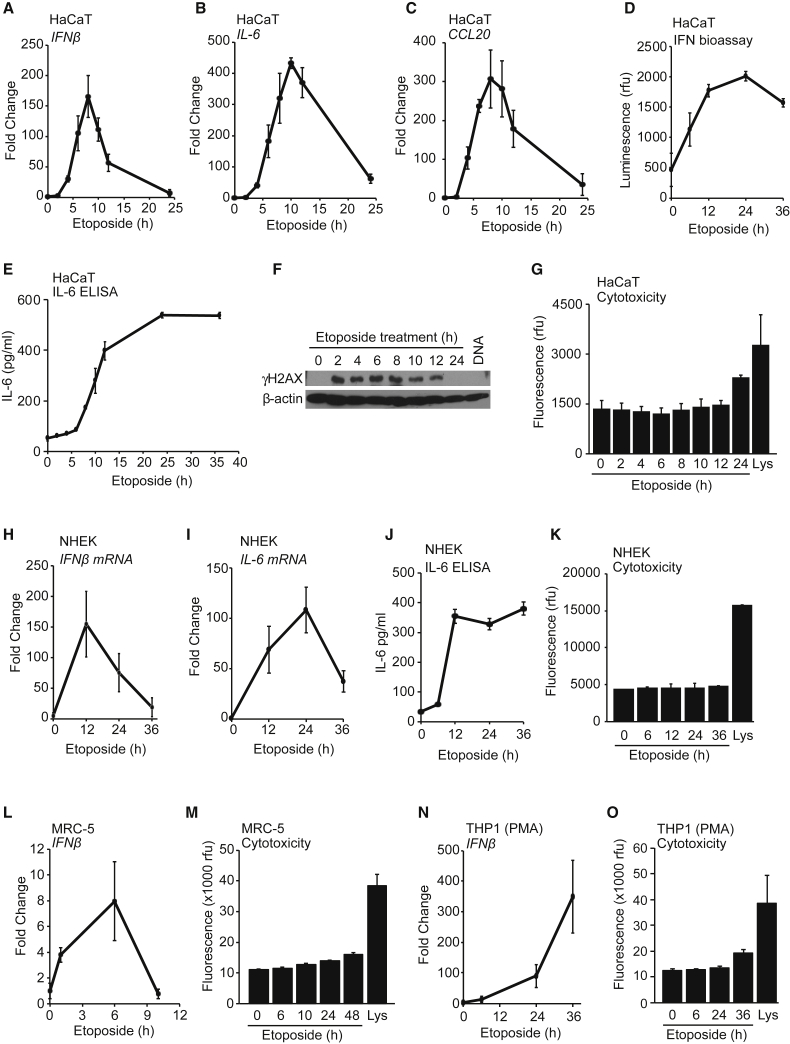
Etoposide-Mediated DNA Damage Induces an Acute Innate Immune Response in Human Cells (A–C) HaCaT keratinocytes were treated with 50 μM etoposide for the times indicated before qRT-PCR analysis of *IFN-β* (A), *IL-6* (B), and *CCL20* (C) mRNA. (D and E) Supernatants from cells treated with 50 μM etoposide were analyzed for secreted type I IFN using a bio-assay (D) or IL-6 protein using ELISA (E). (F) HaCaT cells were treated with 50 μM etoposide for the times indicated or transfected with 1 μg/mL herring testis (HT)-DNA for 6 hr. Phosphorylation of γH2A.X was analyzed by immunoblotting. (G) Cytotoxicity assay of HaCaT cells treated with 50 μM etoposide for the times indicated or lysed (Lys). (H and I) Primary normal human epidermal keratinocytes (NHEKs) from adult donors were treated with 50 μM etoposide for the times indicated before qRT-PCR analysis of *IFN-β* (H) and *IL-6* (I) mRNA. (J) Supernatants from NHEK cells treated as in (H) were analyzed for IL-6 secretion by ELISA. (K) Cytotoxicity assay of NHEK cells treated as in (H) or lysed (Lys). (L) Primary MRC-5 fibroblasts were treated with 50 μM etoposide before qRT-PCR analysis of IFN-β mRNA expression. (M) Cytotoxicity assay of MRC-5 cells treated with 50 μM etoposide or lysed (Lys). (N) PMA-differentiated THP1 cells were stimulated with 50 μM etoposide for indicated times before qRT-PCR analysis of *IFN-β* mRNA. (O) Cytotoxicity assay of THP1 cells treated as in (N) or lysed (Lys). Data are presented as mean values of biological triplicates ± SD. See also Figure S1.

We tested the cell-intrinsic response to DNA damage in human keratinocytes, which, in the outermost layer of our skin, are the first point of contact for many pathogens and are exposed to physical and chemical insults from sunlight or environmental toxins. We find that following treatment with the topoisomerase II poison etoposide, immortalized human HaCaT keratinocytes induce an acute innate immune response that involves the expression of *IFN-β*, the cytokine interleukin-6 (*IL-6*), and the chemokine *CCL20* (Figures 1A–1C). mRNA induction was apparent from 4 hr of treatment and peaking after 8–12 hr (Figures 1A–1C). We also detected the secretion of active type I IFN (Figure 1D) and the subsequent induction of interferon-stimulated genes such as *IRF7* and *IFI16* as a consequence of type I IFN signaling (Figures S1A and S1B). Etoposide treatment also caused the secretion of IL-6 protein (Figure 1E). The transcriptional response to DNA damage correlated with the phosphorylation of histone γH2A.X (Figure 1F) and occurred at time points at which etoposide treatment had not yet caused significant cell death and only a small fraction of cells displayed early signs of apoptosis by Annexin V staining (Figures 1G and S1C).

We detected a similar innate immune response to DNA damage in primary normal human epidermal keratinocytes (NHEKs) from adult donors, involving the expression of *IFN-β*, *IL-6*, and *CCL20* mRNA (Figures 1H, 1I, and S1D) and secretion of IL-6 protein (Figure 1J) at time points at which etoposide treatment did not cause detectable amounts of cell death (Figure 1K). An etoposide-induced innate immune response was also detectable in other cell types, even though the response was more modest in MRC-5 primary human embryonic fibroblasts (Figures 1L, 1M, and S1E–S1G) and started at later time points, after 24–36 hr, in human THP1 monocytes, whether or not they had been differentiated using phorbol 12-myristate 13-acetate (PMA) (Figures 1N, 1O, and S1H–S1L).

### The Innate Immune Response to Etoposide-Induced DNA Damage Involves the DNA Sensing Adaptor STING

**Figure 2:**
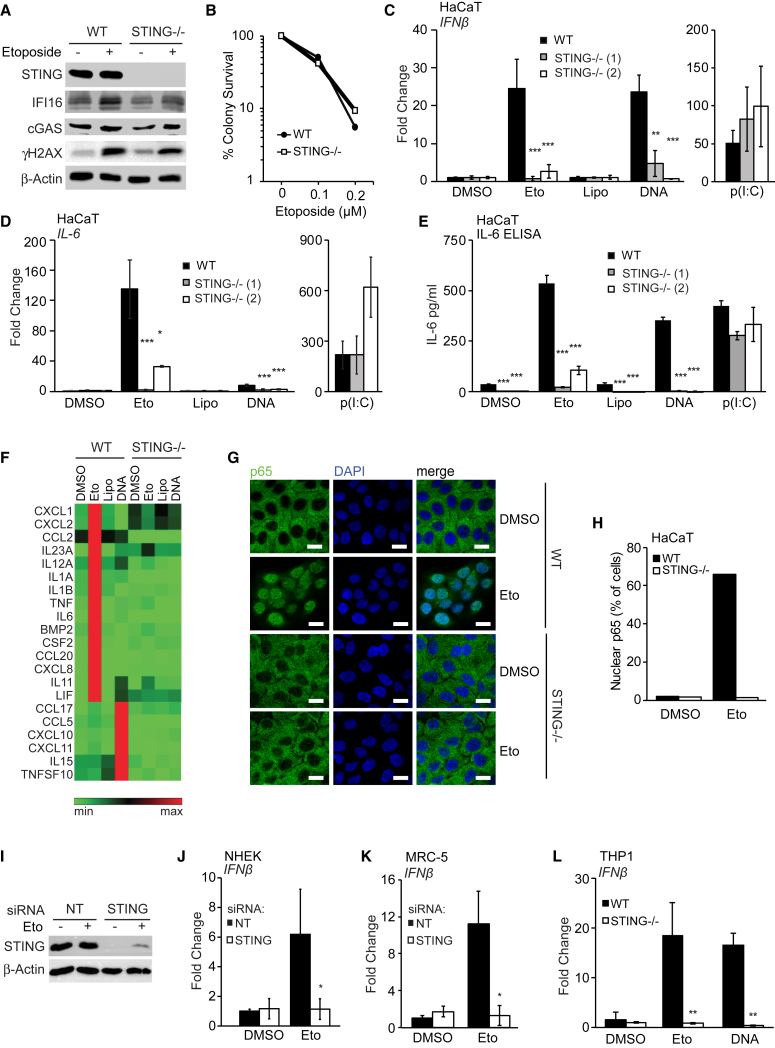
STING Is Required for the Innate Immune Response to Etoposide-Induced DNA Damage (A) Wild-type (WT) and *STING*^−/−^ HaCaT cells were treated with DMSO or 50 μM etoposide for 6 hr, and protein expression was analyzed by immunoblotting. (B) Clonogenic survival assay of WT and *STING*^−/−^ HaCaT cells. Numbers of colonies > 50 cells were counted and expressed as a percentage of untreated control. (C and D) WT HaCaT and two STING^−/−^ clones were treated with DMSO or 50 μM etoposide, mock transfected (Lipo), or transfected with 1 μg/mL HT-DNA or 100 ng/mL poly(I:C) for 6 hr before qRT-PCR analysis of *IFN-β* (C) and *IL-6* (D) mRNA expression. (E) ELISA analysis of IL-6 secretion in supernatants from cells treated as in (C) for 24 hr. (F) qRT-PCR array analysis of cytokine and chemokine expression in WT and *STING*^−/−^ HaCaT cells treated with DMSO, 50 μM etoposide, Lipofectamine, or 1 μg/mL HT-DNA for 6 hr. Shown are genes induced at least 2-fold over controls. (G and H) WT and *STING*^−/−^ HaCaT cells grown on coverslips were treated with 50 μM etoposide for 4 hr and stained for NF-κB p65 (green) and DNA (DAPI, blue) for analysis by confocal microscopy (G) and quantification of p65 nuclear translocation (H). Scale bar, 20 μm. (I and J) NHEKs were treated with non-targeting (NT) or *STING*-targeting siRNA pools for 48 hr before treatment with 50 μM etoposide for 24 hr. STING protein levels were analyzed by immunoblotting (I), and *IFN-β* mRNA expression was quantified by qRT-PCR (J). (K) MRC-5 fibroblasts were treated with non-targeting (NT) or *STING*-targeting siRNA pools for 48 hr before treatment with 50 μM etoposide for 6 hr and analysis of *IFN-β* mRNA by RT-PCR. (L) PMA-differentiated WT and *STING*^−/−^ THP1 cells were stimulated with 50 μM etoposide for 30 hr or 1 μg/mL HT-DNA for 6 hr before qRT-PCR analysis of *IFN-β* mRNA. Data are presented as mean values of biological triplicates ± SD. See also Figures S2 and S3A–S3F.

We tested whether the DNA sensing adaptor STING is involved in the acute innate immune response to etoposide-induced double-strand breaks. HaCaT keratinocytes lacking STING still expressed cGAS and IFI16, displayed unaltered γH2A.X phosphorylation (Figure 2A), and are able to survive as well as wild-type cells after etoposide treatment (Figure 2B). However, STING-deficient cell clones were unable to induce the transcription of *IFN-β* mRNA after etoposide treatment (Figure 2C). As expected, STING-deficient cells were also impaired in their response to transfected DNA but supported *IFN-β* mRNA induction in response to the dsRNA mimic poly(I:C) (Figure 2C). The lack of STING also impaired *IL-6* mRNA expression and IL-6 protein secretion in response to etoposide treatment or DNA transfection, but not following transfection with poly(I:C) (Figures 2D and 2E).

Despite the involvement of STING in both the response to exogenous DNA and the response to DNA damage, the pattern of innate immune gene induction differed between the two stimuli. Etoposide treatment induced higher levels of *IL-6* and *CCL20* mRNA and lower levels of the chemokine *CXCL10* and the IRF3-responsive gene ISG56 than DNA transfection under conditions for which *IFN-β* mRNA induction is comparable (Figures 2C, 2D, and S2A–S2C). Transcription factor activation profiles also differed between the two responses, with the response to DNA damage involving prominent nuclear translocation of the NF-κB subunit p65 and only modest activation of IRF3 (Figures S2D–S2G). We used a PCR array to quantify the expression of more than 80 cytokines and chemokines in HaCaT cells treated with etoposide or transfected DNA for 4 hr. We found that even though the two stimuli induced different cytokine expression profiles, both responses were STING dependent (Figure 2F). The STING-dependent response to etoposide included the expression of genes such as CCL20, which is not strongly induced by conventional DNA-induced STING signaling (Figures 2F, S2C, and S3A). HaCaT cells lacking STING were also unable to support etoposide-induced NF-κB p65 nuclear translocation or phosphorylation (Figures 2G, 2H, and S3B), as well as TBK1 and IRF3 phosphorylation induced by DNA transfection (Figure S3B).

We confirmed the requirement for STING in the innate immune response to double-strand breaks using RNAi in primary NHEKs (Figures 2I, 2J, and S3C), in MRC-5 fibroblasts (Figures 2K, S3D, and S3E), and in THP1 cells lacking STING (Figures 2L and S3F). We conclude that the acute innate immune response to etoposide-induced double-strand breaks involves the STING-mediated activation of NF-κB p65 and the induction of a non-canonical STING-dependent innate immune gene expression program.

### IFI16 Is Required for the Etoposide-Induced Innate Immune Response

IFI16 is a DNA binding protein that cooperates with cGAS in the detection of cytosolic DNA in human keratinocytes and macrophages (Almine et al., 2017, Jønsson et al., 2017). IFI16 has also been described as a tumor suppressor protein, which associates with DNA damage factors such as BRCA1 and p53 and acts to promote cellular senescence (Aglipay et al., 2003, Clarke et al., 2010, Johnstone et al., 2000). Due to its predominantly nuclear localization, we hypothesized that IFI16 may be a good candidate to provide a link between DNA damage and innate immunity when the cell’s own damaged DNA may be sensed as a DAMP.

**Figure 3:**
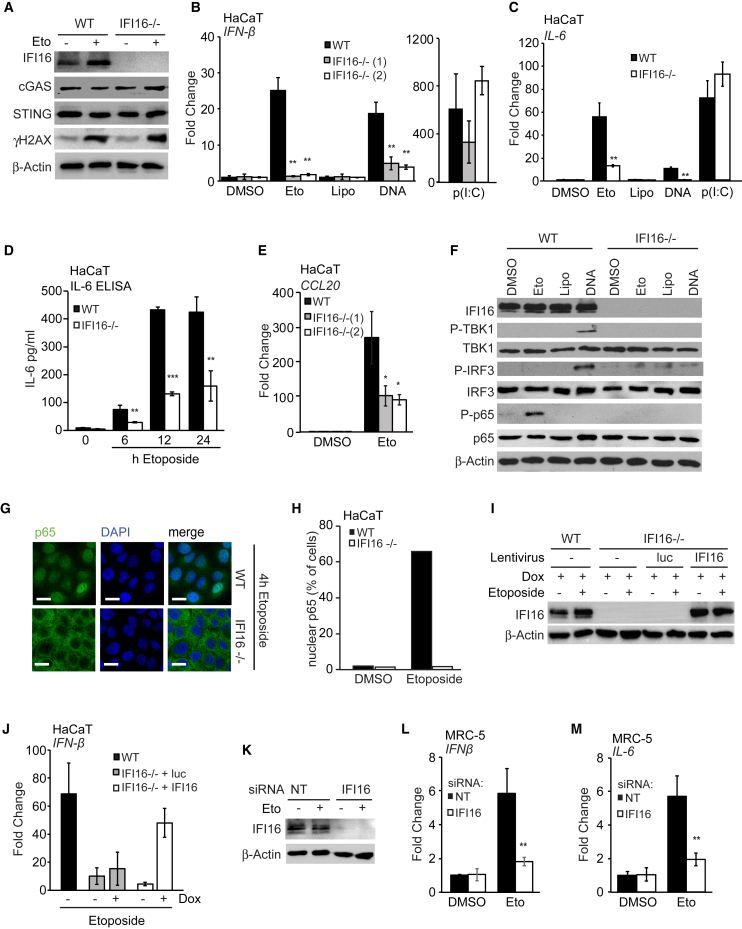
The Innate Immune Response to Etoposide-Induced Damage Involves IFI16 (A) Immunoblotting analysis of WT and *IFI16*^−/−^ HaCaT cells stimulated with 50 μM etoposide or DMSO for 6 hr. (B and C) WT HaCaT cells and two *IFI16*^−/−^ cell clones were treated for 6 hr with DMSO or 50 μM etoposide, mock transfected (Lipo), or transfected with 1 μg/mL HT-DNA or 100 ng/mL poly(I:C). *IFN-β* (B) or *IL-6* (C) mRNA was quantified by qRT-PCR. (D) ELISA analysis of IL-6 protein in supernatants from WT and *IFI16*^−/−^ HaCaT cells treated with 50 μM etoposide for indicated times. (E) qRT-PCR analysis of *CCL20* mRNA in WT and *IFI16*^−/−^ HaCaT cells treated with DMSO or 50 μM etoposide for 6 hr. (F) WT and *IFI16*^−/−^ HaCaT cells were treated as in (B) for 4 hr before analysis of protein expression by immunoblotting. (G) WT and *IFI16*^−/−^ HaCaT cells grown on coverslips were treated with 50 μM etoposide for 4 hr and fixed and stained for p65 (green) and DNA (DAPI, blue). Scale bar, 20 μm. (H) Quantification of p65 nuclear translocation in cells from (G). (I) Immunoblotting analysis of WT HaCaT cells and *IFI16*^−/−^ HaCaT cells reconstituted with lentiviruses for the expression of Luciferase (luc) or IFI16 as indicated. Cells were treated with doxycycline for 24 hr to induce expression and then stimulated with 50 μM etoposide for 6 hr. (J) qRT-PCR analysis of *IFN-β* mRNA in cells treated as in (I) as indicated. (K–M) MRC-5 fibroblasts treated with non-targeting (NT) or *IFI16*-targeting siRNA pools for 48 hr before treatment with 50 μM etoposide or DMSO for 6 hr. IFI16 protein expression was analyzed by immunoblotting (K). *IFN-β* (L) and *IL-6* (M) mRNA levels were analyzed by qRT-PCR. Data are presented as mean values of biological triplicates ± SD. See also Figures S3G–S3L.

IFI16-deficient HaCaT cells were able to support γH2A.X phosphorylation after etoposide treatment (Figure 3A) and displayed a survival advantage over wild-type cells in clonogenic survival assays (Figure S3G). Like STING-deficient cells, two independent HaCaT cell clones lacking IFI16 were unable to induce the expression of *IFN-β* mRNA after etoposide treatment (Figure 3B). As observed previously (Almine et al., 2017), the response to exogenous DNA was also reduced in cells lacking IFI16, while poly(I:C)-induced *IFN-β* mRNA expression was unaffected (Figure 3B). The etoposide-induced secretion of bio-active type I interferons was also impaired in cells lacking IFI16 (Figure S3H), as was the expression of *IL-6* mRNA, the secretion of IL-6 protein, and the expression of *CCL20* mRNA (Figures 3C–3E). IFI16 was also required for the phosphorylation and nuclear translocation of NF-κB following etoposide treatment (Figures 3F–3H).

We confirmed the involvement of IFI16 in the DNA damage-induced innate immune response by reconstituting *IFI16*^−/−^ HaCaT cells with lentivirus for the inducible expression of IFI16 or Luciferase as control. IFI16 re-expression rescued DNA damage-induced *IFN-β* expression (Figures 3I and 3J). The role of IFI16 was confirmed in MRC-5 fibroblasts (Figures 3K–3M and S3I) and in primary NHEKs (Figures S3J–S3L) using RNAi.

### The Acute Innate Immune Response to Etoposide-Induced Damage Is Independent of cGAS and cGAMP

**Figure 4:**
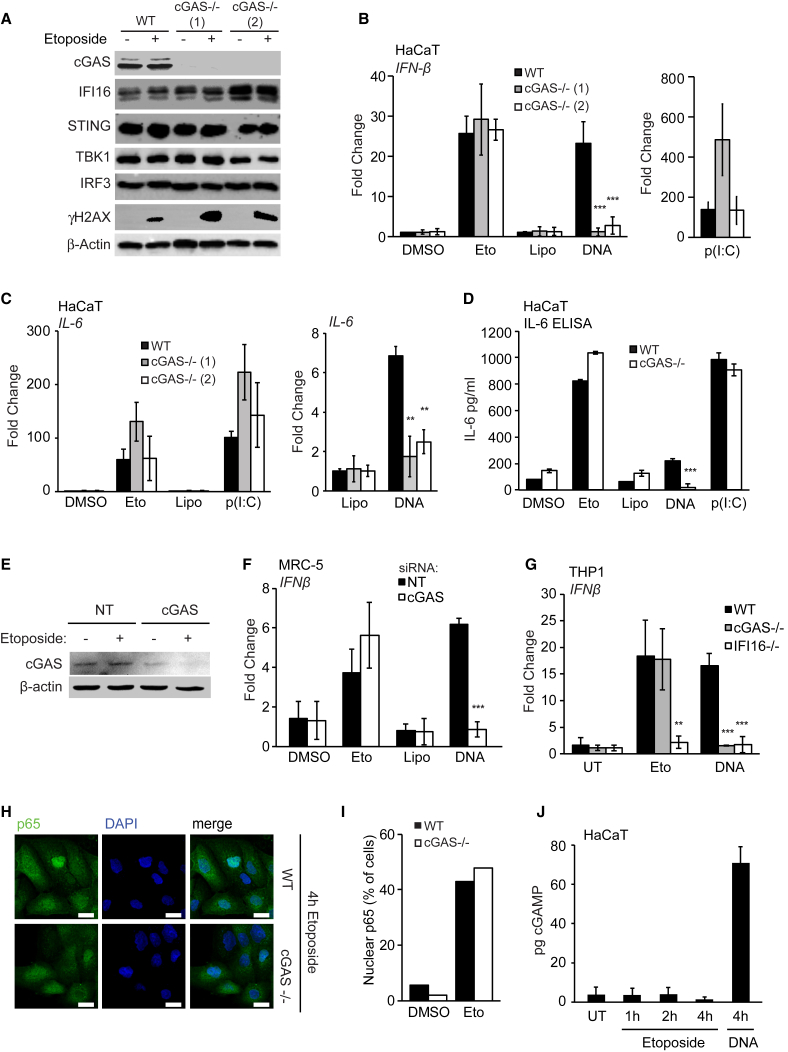
cGAS Is Dispensable for the Early Innate Immune Response to Nuclear DNA Damage (A) Immunoblotting analysis of WT and two *cGAS*^−/−^ HaCaT clones treated with DMSO or 50 μM etoposide for 6 hr. (B and C) WT and *cGAS*^−/−^ HaCaT cells were treated with DMSO or 50 μM etoposide, mock transfected (Lipo), or transfected with 1 μg/mL HT-DNA or 100 ng/mL poly(I:C) for 6 hr before qRT-PCR analysis of *IFN-β* (B) and *IL-6* (C) mRNA expression. (D) IL-6 in supernatants from WT and *cGAS*^−/−^ HaCaT cells treated with 50 μM etoposide quantified by ELISA. (E) MRC-5 fibroblasts were treated with non-targeting (NT) or *cGAS*-targeting siRNA pools for 48 hr before treatment with 50 μM etoposide for 6 hr. cGAS protein expression was analyzed by western blot. (F) qRT-PCR analysis of *IFN-β* mRNA expression in MRC-5 fibroblasts treated with siRNA as in (E) and stimulated with 50 μM etoposide or transfected with 1 μg/mL HT-DNA for 6 hr. (G) PMA-differentiated WT, *cGAS*^−/−^, and *IFI16*^−/−^ THP1 cells were treated with 50 μM etoposide for 30 hr or 1 μg/mL HT-DNA for 6 hr before qRT-PCR analysis of *IFN-β* mRNA. (H) WT and *cGAS*^−/−^ HaCaT cells grown on coverslips were treated with 50 μM etoposide for 4 hr, stained for p65 (green) and DNA (DAPI, blue), and visualized by confocal microscopy. Scale bar, 20 μm. (I) Quantification of p65 translocation from (H). (J) HaCaT cells were treated with 50 μM etoposide for the indicated times or transfected with 1 μg/mL HT-DNA for 4 hr. cGAMP production was quantified by LC-MS. Data are presented as mean values of biological triplicates ± SD. See also Figure S4.

Because IFI16 cooperates with cGAS in the activation of STING, we tested whether cGAS was also required for the early innate immune response following etoposide treatment. cGAS-deficient HaCaT cell clones were still able to support γH2A.X phosphorylation after etoposide treatment (Figure 4A). However, in contrast to the data obtained with IFI16- or STING-deficient cells, cGAS was dispensable for *IFN-β* mRNA induction after etoposide treatment, even though cGAS was essential for *IFN-β* expression after DNA transfection, as expected (Figure 4B). cGAS was also dispensable for the induction of *IL-6* and *CCL20* mRNA after etoposide treatment (Figures 4C and S4A) and the secretion of IL-6 protein measured by ELISA (Figure 4D).

To exclude potential off-target effects or compensatory mechanisms that may have arisen during the generation of cGAS-deficient cell clones, we confirmed the differential roles of cGAS using RNAi in HaCaT keratinocytes (Figure S4B) and in MRC-5 fibroblasts (Figures 4E and 4F). Etoposide-induced IFN-β mRNA expression was impaired in IFI16-deficient THP1 cells, but not cGAS-deficient THP1 cells (Figure 4G), while the response to transfected DNA required the cooperation of both proteins, as described (Jønsson et al., 2017). In agreement with the gene expression data, the absence of cGAS did not affect etoposide-induced NF-κB p65 translocation to the nucleus (Figures 4H and 4I).

### Non-conventional STING Activation after DNA Damage

During the detection of cytosolic DNA, STING is activated by binding the second messenger cGAMP, which causes a conformational change in the STING dimer (Gao et al., 2013). Given that cGAS was not required for the early innate immune response to DNA damage, we tested the possibility that another enzyme in the cell may substitute for cGAS function. We measured the production of endogenous cGAMP in HaCaT cells using a liquid chromatography-mass spectrometry (LC-MS) approach, which allowed us to specifically quantify cGAMP, using c-di-AMP as spike-in control to account for variations arising from sample processing (Figures S4C and S4D). Unlike DNA transfection, Etoposide treatment did not cause any increase in cGAMP production above basal levels (Figures 4J and S4E). This demonstrates that the function of STING during the response to nuclear DNA damage involves its non-canonical activation in a cGAS- and cGAMP-independent manner.

**Figure 5:**
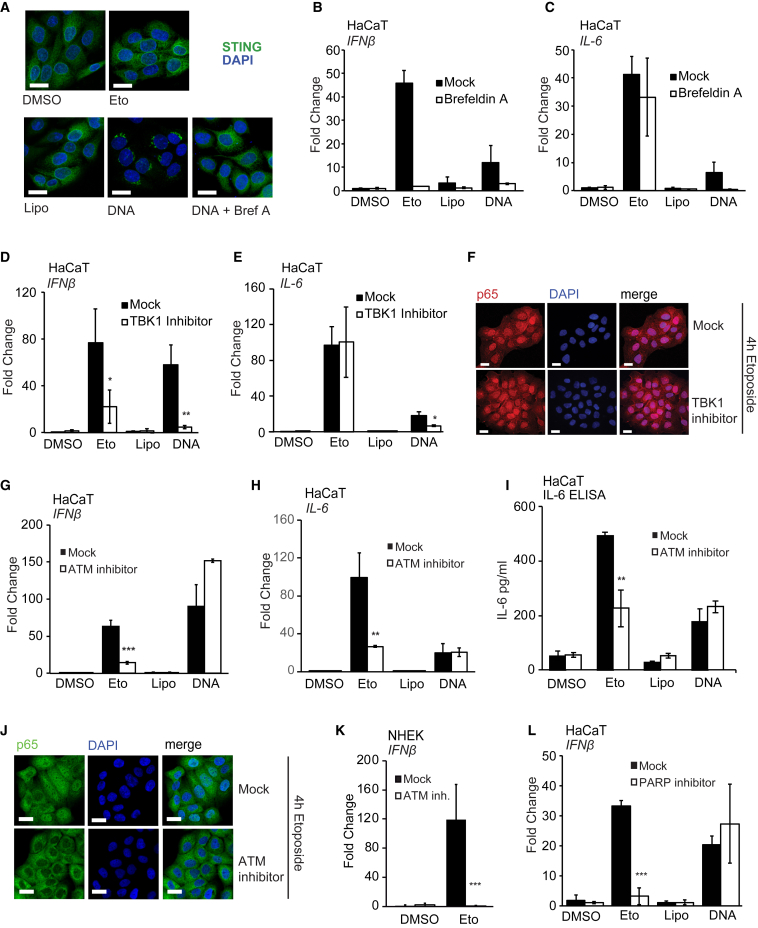
Etoposide-Induced NF-κB Activation Involves DNA Damage Factors, but Not TBK1 Activity (A) HaCaT cells grown on coverslips were pre-treated for 30 min with 3 μg/mL brefeldin A where indicated before stimulation with 50 μM etoposide or transfection of 1 μg/mL HT-DNA. Cells were fixed and stained for STING (green) and DNA (DAPI, blue). Scale bar, 20 μm. (B and C) HaCaT cells were pre-treated for 30 min with 3 μg/mL brefeldin A before treatment with 50 μM etoposide or DMSO, mock transfection (Lipo), or transfection of 1 μg/mL HT-DNA for 6 hr. *IFN-β* (B) and *IL-6* (C) mRNA expression was analyzed by qRT-PCR. (D and E) HaCaT cells were pre-treated for 1 hr with 2 μM TBK1 inhibitor MRT67307 and stimulated as in (B) before qRT-PCR analysis of *IFN-β* (D) and *IL-6* (E) mRNA expression. (F) HaCaT cells grown on coverslips were pre-treated with 2 μM TBK1 inhibitor MRT67307 for 1 hr before 4 hr of stimulation with 50 μM etoposide. Cells were fixed and stained for p65 (red) and DNA (DAPI, blue). Scale bar, 20 μm. (G and H) HaCaT cells were pre-treated for 1 hr with 10 μM ATM inhibitor KU55933 and stimulated as in (B). *IFN-β* (G) and *IL-6* (H) mRNA expression was quantified by qRT-PCR. (I) ELISA analysis of IL-6 secretion in supernatants from cells treated as in (G) and stimulated for 24 hr. (J) HaCaT cells grown on coverslips were pre-treated for 1 hr with 10 μM KU55933 before 4 hr of stimulation with 50 μM etoposide. Cells were fixed and stained for p65 (green) and DNA (DAPI, blue). Scale bar, 20 μm. (K) qRT-PCR analysis of IFN-β mRNA expression in NHEK cells pre-treated for 1 hr with 10 μM KU55933, followed by treatment with 50 μM etoposide for 24 hr. (L) qRT-PCR analysis of *IFN-β* mRNA in HaCaT cells pre-treated for 1 hr with 10 μM PARP inhibitor PJ34 before treatment as in (B) for 6 hr. Data are presented as mean values of biological triplicates ± SD. See also Figure S5.

During conventional DNA sensing, STING translocates from the ER to peri-nuclear foci and gets phosphorylated by TBK1 at Serine 366 (Dobbs et al., 2015, Liu et al., 2015). Etoposide treatment did not cause any detectable STING phosphorylation (Figure S5A) or STING translocation to characteristic foci (Figure 5A). To test whether STING translocation is dispensable or whether some low level of translocation occurs, which is not apparent by confocal microscopy, we inhibited STING translocation using brefeldin A (Figure 5A). Pre-treatment with brefeldin A inhibited the expression of *IFN-β* in response to both etoposide treatment and DNA transfection (Figure 5B). However, although *IL-6* mRNA expression after DNA transfection also required STING trafficking, trafficking was not required for DNA damage-induced *IL-6* expression (Figure 5C). Similar results were obtained with a TBK1 inhibitor (Clark et al., 2009), which impaired etoposide-induced *IFN-β* mRNA expression, but not *IL-6* expression (Figures 5D and 5E), while both *IFN-β* and *IL-6* mRNA expression were TBK1 dependent in response to cytosolic DNA, as described (Abe and Barber, 2014). TBK1 function was also dispensable for the etoposide-induced nuclear translocation of NF-κB p65 (Figures 5F and S5B). This suggests that low levels of STING trafficking and TBK1 activation must occur after etoposide-induced damage and contribute to the induction of *IFN-β* mRNA under these conditions. The expression of NF-κB-dependent genes such as *IL-6*, however, proceeds independently of STING trafficking and TBK1 activity.

### The DNA Damage Factors ATM and PARP-1 Are Required for the Innate Immune Response to Double-Strand Breaks

The early activation of NF-κB in response to double-strand breaks has been shown to involve the DNA damage kinase ATM and PARP-1 (Hinz et al., 2010, Miyamoto, 2011, Stilmann et al., 2009, Wu et al., 2010). Thus, we tested whether these factors are also involved in the STING-dependent innate immune response to DNA damage that we observe in human keratinocytes. We found that inhibition of ATM impaired the expression of *IFN-β* and *IL-6* mRNA after DNA damage and the secretion of IL-6 protein in HaCaT keratinocytes (Figures 5G–5I). ATM activity was not required for the induction of *IFN-β* and *IL-6* following DNA transfection (Figures 5G–5I), highlighting the differences between these two STING-dependent responses. ATM was also required for the nuclear translocation of NF-κB p65 (Figures 4J and S5C) and p65 phosphorylation after etoposide treatment (Figure S5D). A role for ATM was confirmed in NHEK (Figures 5K, S5E, and S5F) and in MRC-5 fibroblasts (Figures S5G–S5I). Like ATM, PARP-1 was also specifically required for the induction of *IFN-β* and *IL-6* expression in response to nuclear DNA damage but was dispensable for the detection of cytosolic DNA (Figures 5L and S5J).

### ATM-Dependent Assembly of a Non-canonical Signaling Complex Containing STING

**Figure 6:**
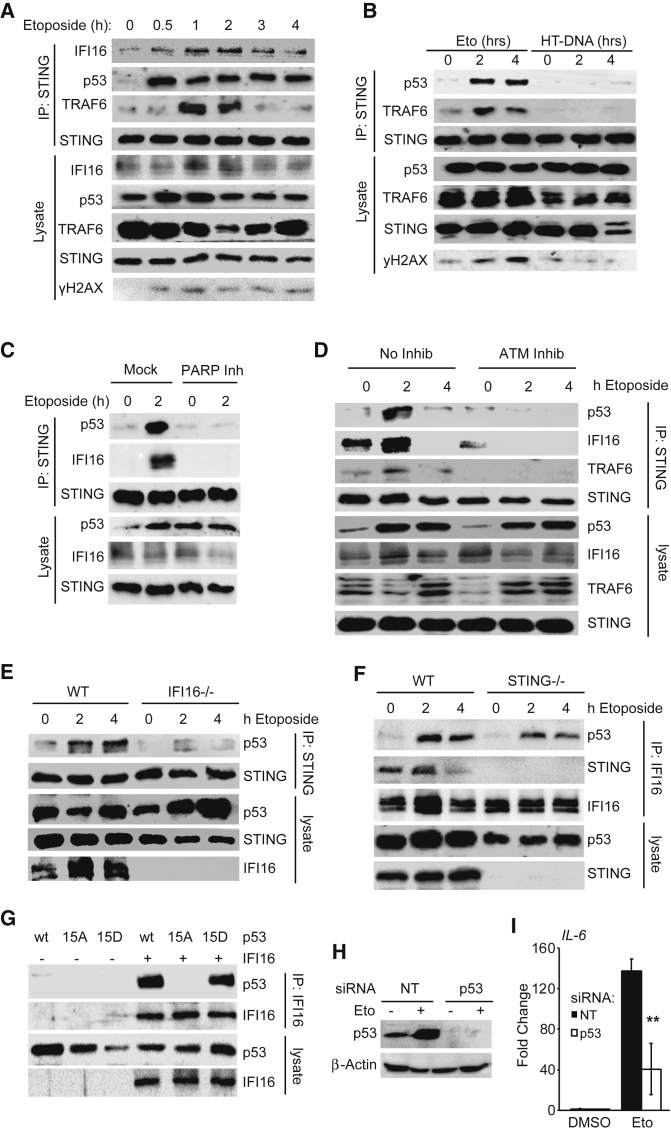
Nuclear DNA Damage Results in the Assembly of a Non-canonical Signaling Complex Containing STING (A) Immunoprecipitation of STING from HaCaT cells treated with 50 μM etoposide for the indicated times. Immunoprecipitates (IPs) and whole-cell lysates were analyzed by immunoblotting. (B) Immunoblotting analysis following immunoprecipitation of STING from HaCaT cells treated with 50 μM etoposide or transfected with 1 μg/mL HT-DNA as indicated. (C) Immunoprecipitation of STING from HaCaT cells pre-treated for 1 hr with 10 μM PARP inhibitor PJ34, followed by treatment with 50 μM etoposide for 2 hr. (D) Immunoprecipitation of STING from HaCaT cells pre-treated for 1 hr with 10 μM ATM inhibitor KU55933 followed by treatment with 50 μM etoposide. (E) Immunoprecipitation of STING from WT and *IFI16*^−/−^ HaCaT cells treated with 50 μM etoposide as indicated. (F) Immunoprecipitation of IFI16 from WT and *STING*^−/−^ HaCaT cells treated with 50 μM etoposide as indicated. (G) HEK293T cells transfected with expression constructs for IFI16 and WT p53 or the S15A or S15D p53 mutants as indicated. 24 hr after transfection, IFI16 was immunoprecipitated from lysates. (H) p53 protein levels in HaCaT cells transfected with a non-targeting (NT) or a *p53*-targeting siRNA pool for 48 hr before stimulation with 50 μM etoposide for 6 hr. (I) qRT-PCR analysis of *IL-6* mRNA expression in cells treated as in (H). See also Figure S6.

After detection of nuclear double-strand breaks, ATM and PARP-1 signal inside out to activate TRAF6 and the IKK complex in the cytosol (Miyamoto, 2011). ATM also phosphorylates p53, which has been shown to interact with IFI16 (Liao et al., 2011). To investigate whether non-canonical activation of STING involves these factors, we interrogated STING complex assembly using immunoprecipitation of endogenous STING in etoposide-treated HaCaT cells. We found that the interaction between IFI16 and STING increased transiently after etoposide treatment and p53 and TRAF6 assemble on STING within 30 min and 1 hr of etoposide treatment, respectively (Figure 6A). This complex is specifically induced by DNA damage and does not form when STING is activated by DNA transfection (Figure 6B). Complex formation requires the function of both PARP-1 and ATM (Figures 6C and 6D). The DNA damage-induced STING complex likely forms at the ER where STING resides, because p53, IFI16, and TRAF6 all shuttle between nucleus and cytosol and become enriched in the membrane fraction of cells after etoposide treatment (Figure S6A).

Both IFI16 and p53 assemble on STING soon after etoposide treatment, and interaction between p53 and IFI16 has been shown to be direct (Liao et al., 2011). Using IFI16-deficient HaCaT cells, we observed that IFI16 was required for the recruitment of p53 to STING (Figure 6E), while the absence of STING still permitted the IFI16-p53 interaction (Figure 6F). This suggests that IFI16 forms a complex with p53 and delivers it to STING after DNA damage, in a manner that depends on ATM activity. ATM phosphorylates p53 at several residues, including Serine 15 (Ser15), which results in the activation of p53 (Banin et al., 1998, Canman et al., 1998). To assess the role of p53 Ser15 phosphorylation in complex assembly, we co-expressed IFI16 and p53 in HEK293T cells, using wild-type p53 or an alanine mutant at position 15 (15A), which cannot be phosphorylated, or using a mutant for which Ser15 is substituted with aspartate (15D), which acts as a phospho-mimic (Loughery et al., 2014). Only wild-type p53 and the 15D phospho-mimic were able to interact with IFI16; the 15A mutant was unable to do so (Figure 6G). A similar pattern was observed for the interaction between p53 and STING (Figure S6B), demonstrating that p53 phosphorylation at Ser15 is a requirement for complex formation.

This association of p53 to the STING complex was functionally important for the innate immune response to DNA damage, because p53 depletion by small interfering RNA (siRNA) impaired the expression of *IL-6*, *IFN-β*, and *CCL20* in HaCaT keratinocytes (Figures 6H, 6I, S6C, and S6D). HaCaT cells are spontaneously immortalized and express mutated forms of p53, which are partially functional, but may also have acquired additional functions (Boukamp et al., 1988, Lehman et al., 1993). Thus, we tested p53 function in primary human MRC-5 fibroblasts that express wild-type p53. We found a similar p53 dependence of etoposide-induced *IFN-β* expression in fibroblasts (Figures S6E and S6F). Altogether, our data show that phosphorylation of p53 at Ser15 likely provides a link between ATM activity and assembly of a STING-containing innate immune signaling complex.

### TRAF6 Mediates STING-Dependent NF-κB Activation after DNA Damage

**Figure 7:**
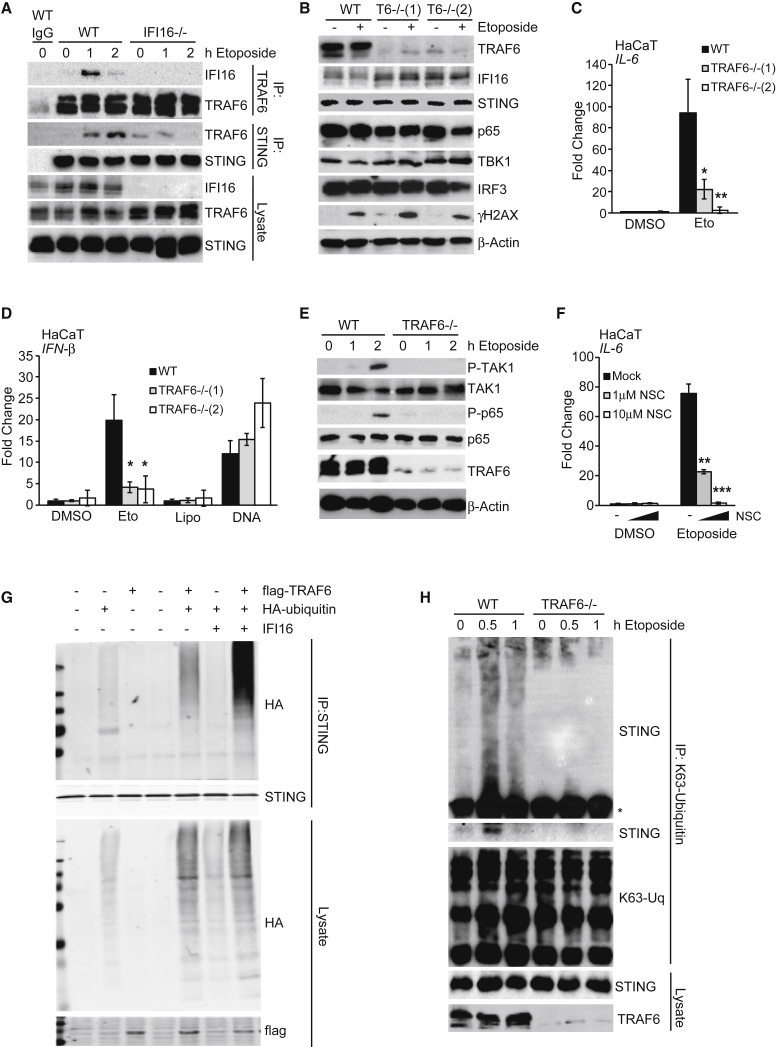
TRAF6 Mediates the K63-Linked Poly-ubiquitylation of STING (A) Immunoprecipitation of TRAF6 and STING from WT and *IFI16*^−/−^ HaCaT cells treated with 50 μM etoposide as indicated. Immunoprecipitates (IP) with immunoglobulin G (IgG) control and input lysates were analyzed by immunoblotting. (B) WT and two *TRAF6*^−/−^ HaCaT clones were treated with 50 μM etoposide for 6 hr, and protein expression was analyzed by immunoblotting. (C) qRT-PCR analysis of *IL-6* mRNA expression in cells treated as in (B). (D) WT and *TRAF6*^−/−^ HaCaT cells were treated with 50 μM etoposide or DMSO, mock transfected (Lipo), or transfected with 1 μg/mL HT-DNA for 6 hr before qRT-PCR analysis of *IFN-β* mRNA. (E) Immunoblotting analysis of WT and *TRAF6*^−/−^ HaCaT cells treated with 50 μM etoposide for the indicated times. (F) HaCaT cells were pre-treated for 1 hr with the indicated concentrations of Ubc13 inhibitor NSC697923 (NSC) before 6 hr of stimulation with 50 μM etoposide. *IL-6* mRNA expression was quantified by qRT-PCR. (G) HEK293T cells were transfected with plasmids for the expression of IFI16, FLAG-tagged TRAF6, and hemagglutinin (HA)-tagged ubiquitin as indicated. 24 hr after transfection, STING was immunoprecipitated, and proteins in immunoprecipitates and input lysates were analyzed by immunoblotting. (H) Immunoprecipitation of K63-linked ubiquitin chains from WT and *TRAF6*^−/−^ HaCaT cells treated with 50 μM etoposide for the times indicated. Higher molecular weight forms of modified STING are visualized by gradient SDS-PAGE above the antibody heavy chain (^∗^), top panel, together with the association of unmodified STING, lower panel. See also Figure S7.

We next investigated the role of TRAF6 in the STING complex. We found that TRAF6 transiently interacted with IFI16 in etoposide-treated HaCaT cells, and IFI16 was required for the etoposide-induced recruitment of TRAF6 to the STING complex (Figure 7A). TRAF6 has an important function in this response, because two TRAF6-deficient HaCaT cell clones were able to support γH2A.X phosphorylation after etoposide treatment (Figure 7B) but were impaired in their ability to induce *IL-6*, *IFN-β*, and *CCL20* mRNA after DNA damage, while the response to exogenous DNA was unaffected (Figures 7C, 7D, and S7A).

TRAF6 is a component of many innate immune signaling complexes, where it catalyzes the formation of K63-linked ubiquitin chains, which then mediate the recruitment of TAB2/3 and TAK1, leading to activation of the IKK complex and phosphorylation of NF-κB p65 (Walsh et al., 2015). We also observe TRAF6-dependent TAK1 and p65 phosphorylation after DNA damage (Figure 7E). Inhibition of the E2 enzyme Ubc13, which works with TRAF6 to catalyze K63-linked ubiquitin chain formation, impaired the etoposide-induced expression of *IL-6*, *IFN-β*, and *CCL20* (Figures 7F, S7B, and S7C), showing that the assembly of K63-linked ubiquitin chains is an important feature of this response.

TRAF6, but not TRAF2 or TRAF3, was able to cause the assembly of ubiquitin chains on a STING complex when overexpressed (Figure S7D), and this function was enhanced in the presence of IFI16 (Figure 7G). The TRAF6-dependent K63-linked ubiquitylation of endogenous STING could be observed in HaCaT cells within 1 hr of etoposide treatment (Figure 7H), and unmodified STING transiently associated with a complex containing K63-linked ubiquitin chains in a TRAF6-dependent manner (Figure 7H). We conclude that the IFI16-mediated recruitment of TRAF6 to a DNA damage-induced STING complex leads to the assembly of K63-linked ubiquitin chains on STING, promoting the activation of NF-κB after nuclear DNA damage.

## Discussion

In this work, we describe a non-canonical mode of STING activation, which results in the induction of an acute innate immune response within hours of etoposide-induced damage. This cell-intrinsic response is distinct from the delayed response due to the detection of DNA from cytosolic micronuclei, which involves cGAS and STING (Dou et al., 2017, Glück et al., 2017, Harding et al., 2017, Mackenzie et al., 2017). The early response to double-strand breaks appears to be particularly potent in keratinocytes, where it might serve as an early warning system for DNA damage induced by UV light or environmental toxins or for the presence of nuclear DNA viruses, which may also be detected by the DNA damage machinery (Turnell and Grand, 2012). A role for DNA damage factors and particularly p53 in anti-viral defense and innate immunity has been proposed, because p53 is responsive to type I interferons, can act as a restriction factor for viruses and transposons, and is in turn targeted by multiple viral evasion mechanisms (Levine et al., 2016, Miciak and Bunz, 2016, Muñoz-Fontela et al., 2016). Here, we link p53 activation to innate immunity, with the ATM-mediated phosphorylation of p53 at Ser15 being required for the association of p53 with a STING-containing signaling complex. Because cooperation between p53 and p65 in the transactivation of innate immune genes, including *IL-6* and *CCL20*, has been observed (Lowe et al., 2014), it remains to be investigated whether p53 acts as a transcription factor and/or has a role in NF-κB activation during the transcriptional response to etoposide.

We show that ATM, PARP-1, and IFI16 are important in the assembly of the DNA damage-induced STING complex. This raises important questions about the nature of the stimulus that drives STING activation after the recognition of double-strand breaks. Although the molecular mechanism of double-strand break recognition by ATM and the subsequent phosphorylation of p53 provide a molecular link between DNA damage and complex formation, it is unknown whether IFI16 is also involved in the recognition of DNA damage or replication stress in the nucleus. It is conceivable that IFI16’s ability to bind unchromatinized (naked) stretches of dsDNA (Morrone et al., 2014, Stratmann et al., 2015) may contribute to the recognition of damaged DNA in the nucleus and provide a further DNA damage signal to activate STING.

The cGAS-independent activation of STING that we observe after etoposide-induced damage appears to proceed largely in the absence of the hallmarks of canonical STING activation: we do not detect STING phosphorylation at Serine 366 or translocation to peri-nuclear foci. However, low levels of STING trafficking and TBK1 activation probably occur and are important for the expression of *IFN-β* after etoposide treatment, possibly through the low-level activation of IRF3, which is required for the transactivation of the *IFN-β* promoter. The expression of NF-κB-dependent genes such as *IL-6*, however, can proceed in the absence of STING trafficking and TBK1 function, highlighting that STING undergoes a qualitatively different mode of activation under these circumstances.

We find that although both DNA damage and DNA transfection activate *IFN-β* induction, the pattern of transcription factor activation differs markedly, with etoposide treatment showing a greater extent of NF-κB activation than cytosolic DNA sensing, which predominantly activates IRF3. Hence, both treatments result in only partially overlapping patterns of STING-dependent gene induction. After DNA damage, STING-dependent NF-κB p65 activation is accomplished through the recruitment of the E3 ubiquitin ligase TRAF6. We provide evidence that TRAF6 catalyzes the assembly of K63-linked ubiquitin chains on STING, a function that is promoted by IFI16. Although TRAF6 also has ubiquitin ligase-independent roles in innate immune signaling (Strickson et al., 2017), it is likely that the assembly of K63-linked poly-ubiquitin chains on STING causes the recruitment of TAB2/3, TAK1, NEMO, and IKKα/β, in analogy to its role in NF-κB activation in other innate immune signaling cascades (Walsh et al., 2015).

The intense study of STING in DNA sensing has shown that STING is subject to a multitude of post-translational modifications, including modification with K63-, K48-, K27-, and K11-linked ubiquitin chains (Chiang and Gack, 2017, Hu et al., 2016, Mukai et al., 2016). The precise molecular function of these modifications in the regulation of STING function has not yet been elucidated, but it is clear that several post-translational modifications of STING act together to fine-tune STING activity and allow for an appropriate and transient response. Any emerging alternative modes of STING activation are likely to be regulated by a similarly complex network of STING interaction partners and post-translational modifications that act in a stimulus- and time-dependent manner.

Studies show that the DNA damage-induced activation of NF-κB is only one example of novel downstream signaling responses that can be activated by STING. It has been shown that in addition to its now well-defined role in the IFN response, STING can promote autophagy (Watson et al., 2012, Watson et al., 2015), apoptosis (Gulen et al., 2017), ER stress (Moretti et al., 2017), and potassium efflux leading to NLRP3-mediated inflammasome activation (Gaidt et al., 2017). Although most signaling functions of STING involve cGAS and cGAMP, instances of cGAS-independent STING signaling have been described, for instance, after the recognition of viral membrane fusion (Holm et al., 2016) and after recognition of RNA ligands by RIG-I and MDA5 (Franz et al., 2018). The activation of STING by RIG-I-like receptors (RLRs) induces yet another downstream response in limiting the translation of viral mRNAs that, like the response to DNA damage, occurs in the absence of detectable STING translocation and phosphorylation (Franz et al., 2018). It remains to be determined how STING is activated under these conditions and how the mode of STING activation mediates the choice of one STING-dependent signaling pathway over another. Our work provides evidence for the notion that STING is not solely an adaptor in the cytosolic DNA sensing response but rather acts a signaling hub that integrates input signals from several sensors in the cell and shapes the resulting cell-intrinsic response, depending on the type of threat.

## STAR★Methods

### Key Resources Table

**Table:** 

REAGENT or RESOURCE	SOURCE	IDENTIFIER
**Antibodies**		

STING (Western, IP)	Cell Signaling	Cat#13647; RRID: AB_2732796
Phospho(Ser366)-STING (Western)	Cell Signaling	Cat#85735
p53 (Western)	Cell Signaling	Cat#9282; RRID: AB_10693944
Phospho(Ser139)-H2A.X (Western)	Cell Signaling	Cat#2577; RRID: AB_2118010
NF-κBp65 (Western, IF)	Cell Signaling	Cat#6956; RRID: AB_10828935
Phospho(Ser536)-NF-κBp65 (Western)	Cell Signaling	Cat#3033; RRID: AB_331284
TBK1 (Western)	Cell Signaling	Cat#3504; RRID: AB_2255663
Phospho(Ser172)-TBK1 (Western)	Cell Signaling	Cat#5483; RRID: AB_10693472
IRF3 (Western, IF)	Cell Signaling	Cat#11904; RRID: AB_2722521
Phospho(Ser396)-IRF3 (Western)	Cell Signaling	Cat#4947; RRID: AB_823547
TRAF6 (Western, IP)	Cell Signaling	Cat#8028; RRID: AB_10858223
HA-tag (Western)	Cell Signaling	Cat#2367; RRID: AB_331789
K63-linked Ubiquitin (Western, IP)	Cell Signaling	Cat#5621; RRID: AB_10827985
IFI16 (N-terminal) (Western)	Santa Cruz	Cat#Sc-8023; RRID: AB_627775
IFI16 (C-terminal) (Western, IP)	Santa Cruz	Cat#Sc-6050; RRID: AB_648739
cGAS (Western)	Sigma Prestige	Cat#HPA031700; RRID: AB_10601693
Histone 3	Cell Signaling	Cat#4499; RRID: AB_10544537
GAPDH	Santa Cruz	Cat#Sc-166545; RRID: AB_2107299
AIF	Cell Signaling	Cat#5318; RRID: AB_10634755
β-actin (Western)	Sigma	Cat#A2228; RRID: AB_476697
Anti-mouse-HRP (Western)	Cell Signaling	Cat#7076; RRID: AB_330924
Anti-rabbit-HRP (Western)	Cell Signaling	Cat#7074; RRID: AB_2099233
Anti-goat-HRP (Western)	Santa Cruz	Cat#Sc-2020; RRID: AB_631728
Anti-mouse-AF647 (IF)	Thermo Fisher	Cat#A21236; RRID: AB_2535805
Anti-mouse-AF488 (IF)	Thermo Fisher	Cat#A11029; RRID: AB_2534088
Anti-rabbit-AF488 (IF)	Thermo Fisher Scientific	Cat#A11034; RRID: AB_2576217

**Bacterial and Virus Strains**		

pLVX-TetOne-Puro-Luc	Clontech	Cat#631849
pLVX-TetOne-Puro-IFI16	This paper	N/A
NovaBlue Competent cells	Novagen	Cat#69284

**Chemicals, Peptides, and Recombinant Proteins**		

Etoposide	Sigma	Cat#E1383
Iodoacetamide	Sigma	Cat#I1149
DNase I	Thermo Scientific	Cat#EN0525
Lipofectamine 2000	Invitrogen	Cat#11668500
GeneJuice	Merck	Cat#70967
Herring Testis DNA	Sigma	Cat#D6898
poly(I:C)	Sigma	Cat#P9582
Polybrene	Sigma	Cat#H9286
Puromycin	Sigma	Cat#P8833
Doxycycline	Sigma	Cat#D9891
DNA Quick Extract Solution	EpiBio	Cat#QE09050
Lightcycler FastStart DNA Master SYBR Green 1	Roche	Cat#03003230001
SYBR Green PCR Master Mix	Applied Biosystems	Cat#43-643-46
Protein G Sepharose beads 4 Fast Flow	GE Healthcare	Cat#GE17-0618-01
Clarity ECL Western Blotting Substrate	Bio-Rad	Cat#1705061
Clarity Max ECL Western Blotting Substrate	Bio-Rad	Cat#1705062
Giemsa stain	Sigma	Cat#GS500
MOWIOL 488	Calbiochem	Cat#475904
ATM Inhibitor KU55933	Santa Cruz	Cat#sc-202963
TBK1 Inhibitor MRT67307	MRC Protein Phosphorylation and Ubiquitylation Unit, University of Dundee	N/A
PARP Inhibitor PJ34	Sigma	Cat#P4365
Brefeldin A	eBioscience	Cat#00-4506-51
Ubc13 inhibitor NSC697923	Sigma	Cat#SML0618

**Critical Commercial Assays**		

CellTox Green Cytotoxicity Assay	Promega	Cat#G8741
Annexin V apoptosis detection kit	eBioscience	Cat#88-8005
Cell Fractionation Kit	Cell Signaling	Cat#9038
Human IL-6 DuoSet ELISA	R&D Systems	Cat#DY206-05
One-Glo Luciferase Reporter Assay System	Promega	Cat#E6110
Lightcycler480 High Resolution Melting master mix	Roche	Cat#04909631001
EZNA total RNA Kit	Omega Bio-TEK	Cat#R6834-02
iScript cDNA Synthesis Kit	Bio-Rad	Cat#170-8891
RT2 First Strand Mix	QIAGEN	Cat#330401
RT2 Profiler PCR Array Human Cytokines and Chemokines	QIAGEN	Cat#PAHS-150ZF

**Deposited Data**		

Raw data of images	This paper, Mendeley Data	https://doi.org/10.17632/5vxm8rptk2.1

**Experimental Models: Cell Lines**		

Human: cell line HaCaT	DKFZ	Cat#300493
Human: IFI16-deficient HaCaT cells BF4	Almine et al., 2017	N/A
Human: IFI16-deficient HaCaT cells 4-11	Almine et al., 2017	N/A
Human: STING-deficient HaCaT cells 3-B3	This Paper	N/A
Human: STING-deficient HaCaT cells 3-C8	This Paper	N/A
Human: cGAS-deficient HaCaT cells 1-A8	Almine et al., 2017	N/A
Human: cGAS-deficient HaCaT cells 2-A6	This Paper	N/A
Human: TRAF6-deficient HaCaT cells 22	Strickson et al., 2017	N/A
Human: TRAF6-deficient HaCaT cells 44	Strickson et al., 2017	N/A
Human: HEK293 cells expressing pGreenFire-ISRE	Dr. Jan Rehwinkel	N/A
Human: cell line NHEK	Lonza	Cat#192627
Human: cell line MRC-5	Dr. Michael Nevels	N/A
Human: cell line THP1	ECACC; Dr. Martin Jakobsen	ECACC 88081201; Jønsson et al., 2017
Human: IFI16-deficient THP1 cells	Jønsson et al., 2017	N/A
Human: STING-deficient THP1 cells	Jønsson et al., 2017	N/A
Human: cGAS-deficient THP1 cells	Jønsson et al., 2017	N/A

**Oligonucleotides**		

Non-targeting siRNA pool	GE Dharmacon	Cat#D-001810-10-05
IFI16-targeting siRNA pool	GE Dharmacon	Cat#L-020004-00-0005
STING-targeting siRNA pool	GE Dharmacon	Cat#L-024333-02-0005
cGAS-targeting siRNA pool	GE Dharmacon	Cat#L-015607-02-0005
p53-targeting siRNA pool	GE Dharmacon	Cat#L-003329-00-0005
See Table S1 for qRT-PCR primer sequences	N/A	N/A

**Recombinant DNA**		

pcDNA3: p53 WT	Loughery et al., 2014	Addgene 69003
pcDNA3: p53 S15A	Loughery et al., 2014	Addgene 69004
pcDNA3: p53 S15D	Loughery et al., 2014	Addgene 69005
pcDNA3.1: IFI16-Flag	This Paper	N/A
pcDNA3.1: STING-Flag	DR L. Jin, Albany Medical Centre	N/A
HA-Ubiquitin	A. Mansell, Monash University	N/A
TRAF2	Tularik, San Francisco	N/A
TRAF3	Tularik Inc., San Francisco	N/A
TRAF6	Tularik Inc., San Francisco	N/A

**Software and Algorithms**		

Odyssey imaging system	LI-COR Biosciences	LI-COR Biosciences
Image Lab	Bio-Rad	http://www.bio-rad.com/en-uk/product/image-lab-software?ID=KRE6P5E8Z
Zen Microscope software	Zeiss	https://www.zeiss.com/microscopy/int/products/microscope-software/zen.html
OMERO	University of Dundee & Open Microscopy Environment	http://www.openmicroscopy.org/omero/
Magellan Data Analysis software	Tecan	https://lifesciences.tecan.com/products/software/magellan_data_analysis_software
CytExpert	Beckman Coulter	https://www.beckman.com/coulter-flow-cytometers/cytoflex/cytexpert
Lightcycler software 4.1	Roche	Cat#04898915001
SABiosciences PCR Array Analysis	QIAGEN	http://dataanalysis.sabiosciences.com/pcr/arrayanalysis.php

### Contact for Reagent and Resource Sharing

Further information and requests for reagents may be directed to and will be fulfilled by the Lead Contact, Leonie Unterholzner (l.unterholzner@lancaster.ac.uk).

### Experimental Model and Subject Details

#### Cell culture

Immortalized human HaCaT keratinocytes, primary MRC-5 human fibroblasts and HEK293T cells were grown in DMEM (Life Technologies) supplemented with 10% Fetal Bovine Serum (FBS; Sigma) and 50μg/mL Gentamycin (Life Technologies). Primary normal human dermal keratinocytes from adult donors (NHEK; Lonza) were grown in KGM-Gold Keratinocyte Basal Medium supplemented with KGM-Gold SingleQuots (Lonza). THP1 cells were grown in RPMI 1640 (Life Technologies) supplemented with 10% FBS and 50 μg/mL Gentamycin, and differentiated with 100nM PMA for 24h where indicated.

THP1 cells lacking STING, IFI16 or cGAS were generated using CRISPR/Cas9 (Jønsson et al., 2017). HaCaT cell clones lacking TRAF6 were generated using Cas9 nickase (Strickson et al., 2017). *IFI16*^*−/−*^ HaCaT cells were generated using TALENs as described (Almine et al., 2017). HaCaT cells lacking cGAS or STING were generated using CRISPR-Cas9 nickase. Plasmids encoding Cas9 nickase and two guide RNAs, were transfected into HaCaT cells using the Neon transfection system (Life Technologies). Cells were selected for 48h with Puromycin, and cell clones were generated by limiting dilution. Cell clones were screened for modifications of the target site, using high resolution melting analysis using LightCycler480 High Resolution Melting master mix (Roche) on a LightCycler 96 system (Roche). Candidate clones were screened by western blotting and immunofluorescence.

### Method Details

#### Plasmids and Transfection

HEK293T cells were transfected using 3μL of GeneJuice (Merck) per 1μg of plasmid DNA. p53 expression plasmids (wild-type, S15A and S15D) were obtained from the David Meek lab, University of Dundee (Loughery et al., 2014). IFI16 expression plasmid was generated by inserting the coding sequence from the *IFI16* B isoform into pcDNA3.1. pcDNA3.1:STING-FLAG was kindly provided by Lei Jin, Albany Medical Centre, the HA-ubiquitin expression construct by A. Mansell, Monash University, and TRAF6 expression plasmids were obtained from Tularik, San Francisco. Transfection for stimulation of cells with exogenous nucleic acids were performed using Lipofectamine 2000 (Thermo Fisher Scientific), using 1 μL Lipofectamine 2000 and 1 μg herring testis (HT) DNA (Sigma) or 100ng poly(I:C) (Sigma) per mL of medium.

Luciferase- and IFI16-expressing lentiviruses derived from vector pLVX-TetOne-Puro (Clontech) were generated in HEK293T cells as described (Harwardt et al., 2016). Lentiviruses were added to cell media with 8μg/mL Polybrene (Sigma) and incubated for 24h, before addition of Puromycin (Sigma) selection medium. Lentiviral gene expression was activated by 24h incubation with 1μg/mL Doxycycline (Sigma).

#### DNA Damaging Agents and Inhibitors

Etoposide and inhibitors were diluted in DMSO. Etoposide (Sigma) was used at a final concentration of 50 μM, unless indicated otherwise. ATM inhibitor KU55933 (Santa Cruz) was used at 10 μM. TBK1 inhibitor MRT67307 was kindly provided by the MRC Protein Phosphorylation and Ubiquitylation Unit, University of Dundee, and used at 2 μM. PARP inhibitor PJ34 (Sigma) was used at 10μM. Brefeldin A (eBioscience) was used at 3μg/mL. Ubc13 inhibitor NSC697923 (Sigma) was used at 1 and 10 μM as indicated.

#### siRNAs

Pools of four individual siRNAs were obtained from GE Dharmacon (SMARTpool: ON-TARGETplus siRNA). Cells were transfected with 3 μL Lipofectamine RNAiMax (Thermo Fisher Scientific) per mL medium and 5nM of non-targeting siRNA pool or *IFI16*-, *STING*-, or *p53*-targeting siRNA for 48h before stimulation of cells.

#### qRT-PCR

RNA was extracted from cells using the EZNA total RNA kit (Omega Bio-TEK), treated with DNase I (Thermo Scientific), and reverse transcribed using the iScript cDNA Synthesis Kit (Bio-Rad Laboratories) according to manufacturer’s instructions. PCR primers were synthesized by Eurofins Genomics, for sequences see Table S1. qRT-PCR amplification was carried out using FastStart Universal SYBR Green master mix (Roche) on a LightCycler 96 realtime PCR instrument (Roche). The cycling program was as follows: initial denaturation at 95°C for 600 s; 40 cycles of 95°C for 10 s and 60°C for 30 s; followed by a melt curve step. Quantification cycle (Cq) for the mRNAs of interest were normalized to *β-actin* reference mRNA and data was expressed as fold change over mock treatment.

For the qRT-PCR array, cDNA was prepared using RT2 First Strand Mix (QIAGEN), and amplified using RT2 SYBR Green qPCR Master Mix (QIAGEN) with the RT2 Profiler PCR Array Human Cytokines and Chemokines (PAHS-150ZF, QIAGEN), according to manufacturer’s instructions. Data was analyzed using SABioscience PCR array analysis software.

#### ELISA and IFN Bio-assay

Secreted interleukin-6 (IL-6) protein in cell supernatants was quantified using the Human IL-6 DuoSet ELISA (R&D Systems) according to manufacturer’s instructions. Results were expressed as pg/mL IL-6 protein in supernatants, based on a standard curve from recombinant IL-6 standards. Bio-active type I IFN in cell supernatants was measured using an IFN bioassay utilizing HEK293 cells stably expressing a pGreenFire-ISRE construct (kindly provided by Jan Rehwinkel). Reporter cells were overlaid with sample cell culture supernatant for 24h, and luminescence was measured using One-Glo Luciferase Assay System (Promega) according to manufacturer’s instructions.

#### Immunoblotting

Cells were lysed in Mammalian Cell Lysis Buffer (50mM Tris-Cl pH 7.5, 1mM EDTA, 1mM EGTA, 1% (v/v) Triton X-100, 1mM sodium orthovanadate, 50mM sodium fluoride, 5mM sodium pyrophosphate, 10mM sodium β-glycerophosphate, 0.27M sucrose, 0.1% (v/v) 2-mercaptoethanol, 0.1mM PMSF, 10 μl/ml Aprotinin) and pre-cleared by centrifugation at 8,000xg for 10 min. In the fractionation experiments, subcellular fractions were separated using the Cell Fractionation kit (Cell Signaling) according to manufacturer’s instructions. Pre-cleared lysates were denatured by boiling in SDS sample buffer (62.5mM Tris-Cl pH 6.8, 2% (w/v) SDS, 10% Glycerol, 0.1% Bromophenol Blue, 50mM DTT). Proteins were separated by SDS-PAGE and transferred onto PVDF membranes (Millipore) using semi-dry transfer (Biometra). Membranes were blocked with 5% (w/v) non-fat milk, or 5% bovine serum albumin (BSA), in 0.1% Tween-20/TBS for 1h. Primary antibodies were used at a dilution of 1:1000. Secondary HRP-coupled antibodies were used at 1:3000. Membranes were developed using Clarity or Clarity Max ECL substrate (Bio-Rad) and imaged on a Chemidoc (Bio-Rad) or Odyssey (LI-COR) imaging system.

#### Co-Immunoprecipitation

Cells were lysed in Mammalian Cell Lysis Buffer (50mM Tris-Cl pH 7.5, 1mM EDTA, 1mM EGTA, 1% (v/v) Triton X-100, 1mM sodium orthovanadate, 50mM sodium fluoride, 5mM sodium pyrophosphate, 10mM sodium β-glycerophosphate, 0.27M sucrose, 0.1% (v/v) 2-mercaptoethanol, 0.1mM PMSF, 10 μL/mL Aprotinin). For the immunoprecipitation of ubiquitin chains, the lysis buffer was supplemented with 50mM iodoacetamide (Sigma). Samples were pre-cleared by centrifugation at 8,000xg for 10 min before incubation with 1 μL of antibodies overnight at 4°C, followed by the addition of a 30 μL protein G beads (Thermo Fisher) for 3h at 4°C. Beads were washed three times with lysis buffer and bound proteins were eluted by boiling in SDS sample buffer for 10 min. A portion of each whole cell lysate was retained as input controls.

#### Confocal Microscopy

For staining of NF-κB p65, cells grown on glass coverslips were fixed for 15 min in 4% paraformaldehyde, and permeabilised for 12 min in 0.5% Triton X-100/PBS. Coverslips were incubated in blocking buffer (5% BSA in 0.05% Tween-20/PBS) for 1h, and stained overnight with primary antibodies (1:600), washed with PBS, and incubated with fluorescently labeled secondary antibodies (1:1500) for 3h. For visualization of STING, cells were fixed in methanol at −20°C, permeabilised, and incubated with blocking buffer containing 5% FBS in 0.05% Tween-20/PBS before incubation with antibodies as above. Coverslips were mounted in MOWIOL 4-88 (Calbiochem) containing 1 μg/mL DAPI (Sigma). Images were taken on a Zeiss LSM880 confocal microscope.

#### Cytotoxicity Assays

Cells were seeded in 96 well plates and treated as indicated. Cyanine Dye and Assay Buffer from CellTox Green Cytotoxicity Assay (Promega) were incubated with cells for 15 min after treatment. Fluorescence was measured using the Infinite M200 PRO (Tecan) plate reader at wavelengths of 485-500nmEx/520-530nmEm.

Cells undergoing apoptosis were quantified using the Annexin V apoptosis detection kit (eBioscience). Cells were treated with Etoposide for the indicated times, then washed and stained with Annexin V and Propidium Iodide according to manufacturer’s instructions. Cells were analyzed by flow cytometry (Cytoflex, Beckman Coulter).

#### Clonogenic Survival Assay

Cells were seeded in 6 well plates and allowed to attach prior to treatment with indicated concentrations of Etoposide. Media was changed after 24 h, and cells were allowed to grow for for a further 14 days. Cells were then washed, fixed, and stained with Giemsa stain (Sigma). The number of colonies containing > 50 cells was counted. Cell viability of untreated cells was treated as 100%.

#### cGAMP Detection by LC-MS

cGAMP detection was carried out as described in Almine et al., 2017. Cells were lysed in cold 80% methanol, and an internal standard of 0.45pmol cyclic di-AMP was added to each sample. Samples then underwent three rounds of butanol:water extraction. Dried samples were resuspended in 1mL H_2_O and purified by solid phase extraction using HyperSep Aminopropyl columns (Thermo Scientific). Columns were washed twice with 2% (v/v) acetic acid and 80% (v/v) methanol. Elution was performed using 4% (v/v) ammonium hydroxide and 80% (v/v) methanol. Samples were resuspended in 50 μL H_2_O for analysis. cGAMP levels were measured by liquid chromatography mass spectrometry (LC-MS) using a TSQ Quantiva interfaced with Ultimate 3000 Liquid Chromatography system (Thermo Scientific), equipped with a porous graphitic carbon column (HyperCarb 30x1mm ID 3 μm; Part No: C-35003-031030, Thermo-Scientific). cGAMP and cyclic di-AMP levels were measured using multiple reaction monitoring mode (MRM) with optimized collision energies. Three transitions (328.03, 343.92 and 522.00) were used to monitor cGAMP and one transition (328.03) was used to detect cyclic di-AMP.

### Quantification and Statistical Analysis

Quantitative data are expressed as mean of biological triplicate samples ± SD. Data were subjected to a multiple t test statistical analysis with the Holm-Sidak method. ^∗^ p < 0.05, ^∗∗^ p < 0.01, ^∗∗∗^ p < 0.001, as indicated in the figure legends.

### Data and Software Availability

Raw data have been deposited to Mendeley Data and are available at https://doi.org/10.17632/5vxm8rptk2.1.

## Supplemental Information

Document S1. Figures S1–S7 and Table S1

Document S2. Article plus Supplemental Information
