# Supplementary material for: Non-canonical Activation of the DNA Sensing Adaptor STING by ATM and IFI16 Mediates NF-κB Signaling after Nuclear DNA Damage
Source: Mol Cell. 2018 Sep 6;71(5):745–760.e5. doi: 10.1016/j.molcel.2018.07.034 (PMC6127031; doi:10.1016/j.molcel.2018.07.034)
Supplement: Document S1. Figures S1–S7 and Table S1 [file mmc1.pdf]

Molecular Cell, Volume *71*

Supplemental Information

Non-canonical Activation of the DNA Sensing Adaptor STING by ATM and IFI16 Mediates NF-κB Signaling after Nuclear DNA Damage

Gillian Dunphy, Sinéad M. Flannery, Jessica F. Almine, Dympna J. Connolly, Christina Paulus, Kasper L. Jønsson, Martin R. Jakobsen, Michael M. Nevels, Andrew G. Bowie, and Leonie Unterholzner

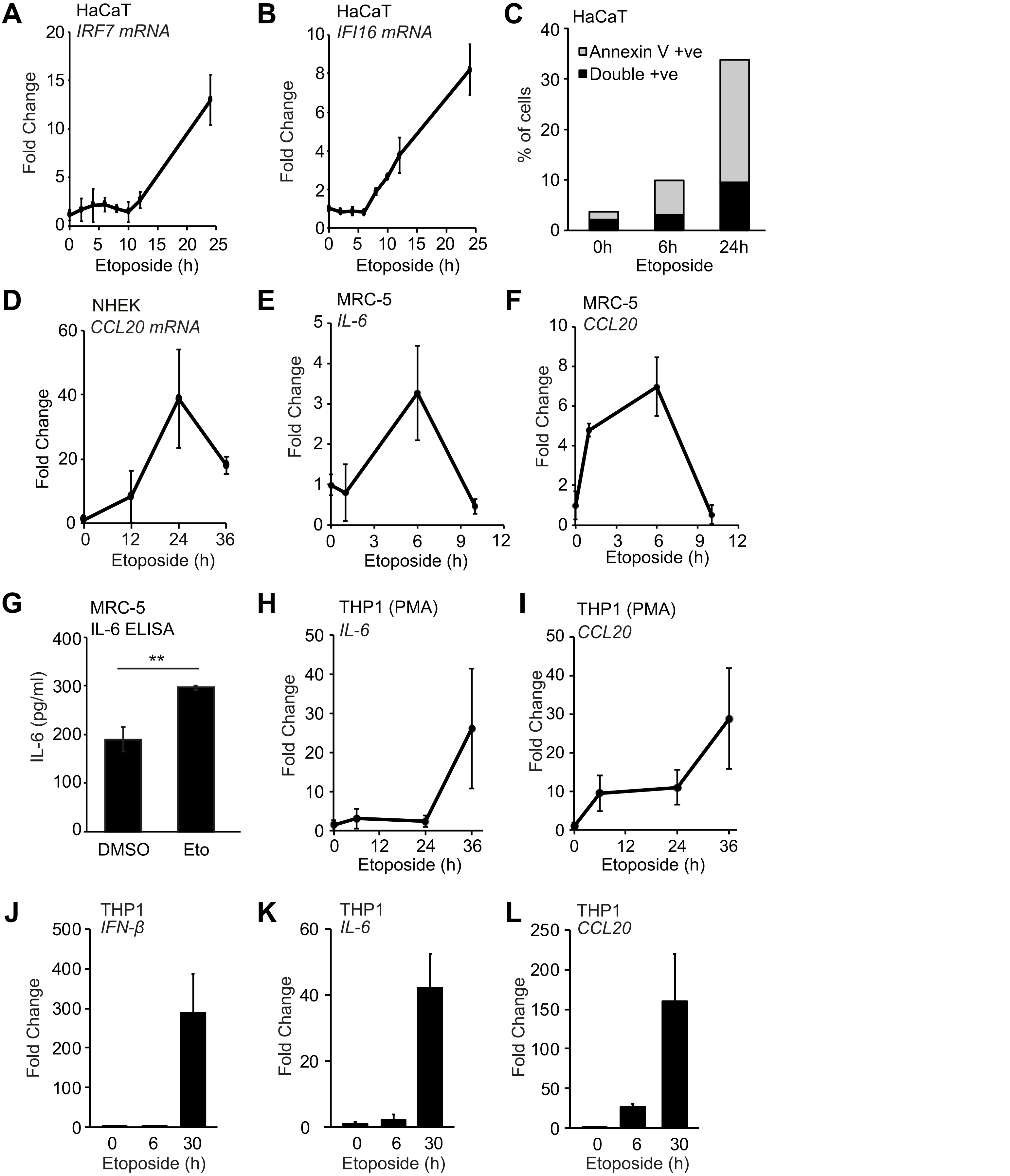

Supplementary Figure 1: Etoposide induces an innate immune response in human cells, Related to Figure 1 **(A, B)** HaCaT cells treated with 50μM Etoposide for indicated times, before qRT-PCR analysis of *IRF7* **(A)**, and *IFI16* **(B)** mRNA. **(C)** WT cells were treated with 50μM Etoposide for indicated times, and stained with Annexin V and Propidium Iodide. Positive staining cells were quantified by flow cytometry. **(D)** qRT-PCR analysis of *CCL20* mRNA in NHEK cells treated with 50μM Etoposide for indicated times. **(E, F)** qRT-PCR analysis of *IL-6* **(E)**, and *CCL20* **(F)** mRNA in Etoposide-treated fibroblasts. **(G)** Supernatants from MRC-5 cells treated with 50μM Etoposide for 24h analysed for protein expression of IL-6 by ELISA. **(H, I)** PMA-differentiated THP1 cells treated with 50μM Etoposide for indicated times. qRT-PCR analysis of *IL-6* **(H)**, and *CCL20* **(I)** mRNA expression. **(J-L)** Undifferentiated THP1 cells stimulated with 50μM Etoposide for indicated times before qRT-PCR analysis of *IFN-β* **(J)**, *IL-6* **(K)**, and *CCL20* **(L)** mRNA expression. Data are presented as average of biological triplicates. Error bars represent SD.

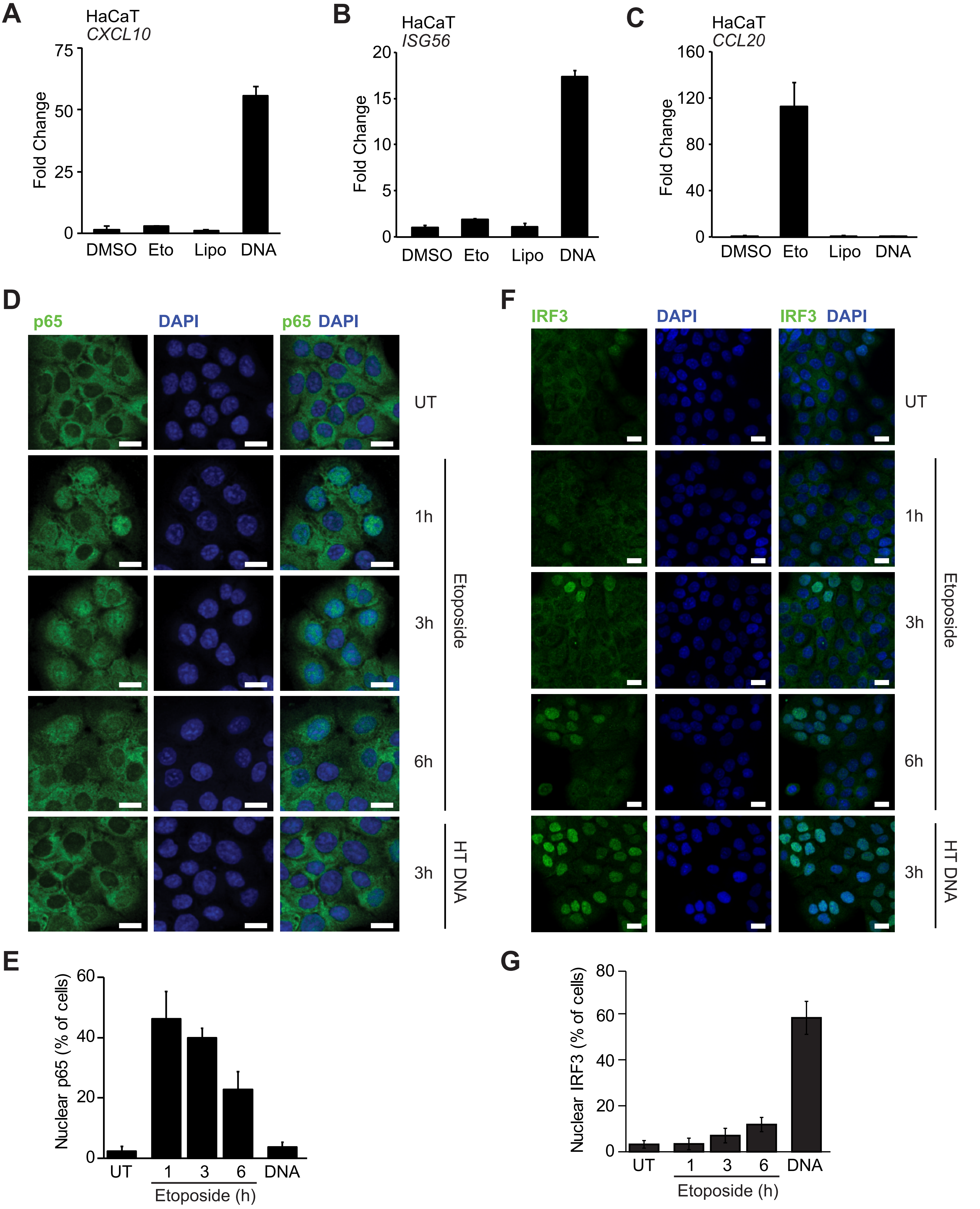

Supplementary Figure 2: Etoposide and DNA transfection induce different gene expression profiles. Related to Figure 2.

(A-C) HaCaT cells were treated with 50μM Etoposide or transfected with 1μg/ml HT-DNA for 6h before lysis for qRT-PCR to analyse expression of *CXCL10* **(A)**, *ISG56* **(B)**, and *CCL20* **(C)** mRNA. **(D-G)** HaCaT cells grown on coverslips were treated with 50μM Etoposide or stimulated with 1μg/ml HT-DNA (HT) for indicated times. Cells were then fixed and stained for DNA (DAPI, blue), p65 (D, green) or IRF3 (F, green). Cells imaged by confocal microscopy were scored for nuclear or cytosolic location of the transcription factors **(E, G)**. At least 200 cells were counted per sample. Scale bar: 20μm. Error bars indicate SD.

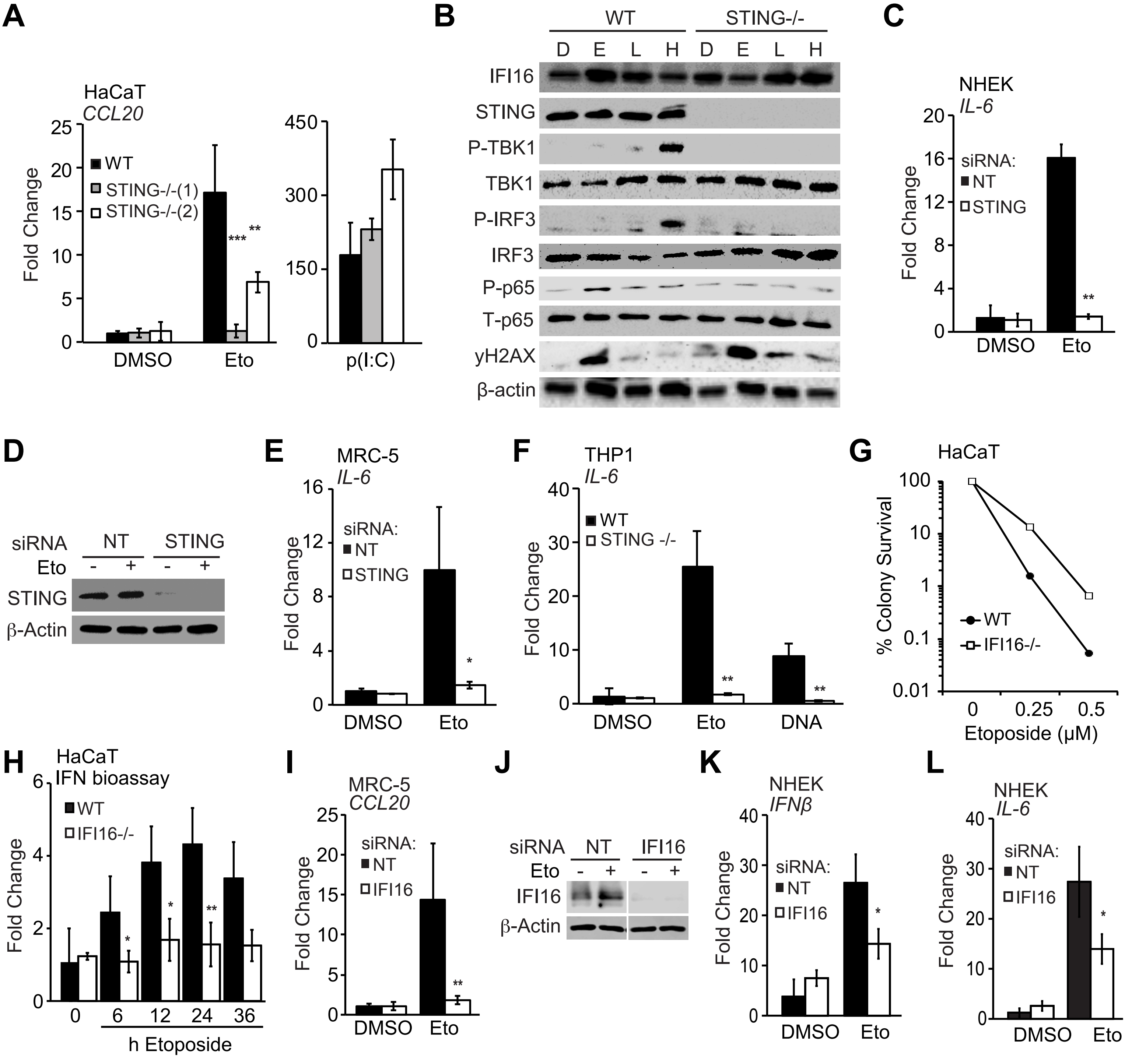

Supplementary Figure 3: STING and IFI16 are required for the innate immune response to Etoposide-induced DNA damage. Related to Figures 2 and 3.

(A) WT and *STING-/-* HaCaT cells were treated with 50μM Etoposide, or transfected with 100ng/ml poly(I:C) for 6h and the expression of *CCL20* mRNA was analysed by qRT-PCR. **(B)** WT and *STING-/-* HaCaTs were treated with DMSO (D), 50μM Etoposide (E), Lipofectamine (L) or transfected 1μg/ml HT-DNA (H) for 6h before analysis of protein expression by immunoblotting. **(C)** qRT-PCR analysis of NHEK cells transfected with a *STING*-targeting or non-targeting (NT) siRNA pool for 48h, followed by treatment with 50μM Etoposide for 6h. **(D, E)** MRC-5 fibroblasts were treated as in (C) before analysis of protein expression by immunoblotting **(D)** and qRT-PCR analysis of *IL-6* mRNA **(E)**. **(F)** PMA-differentiated WT and STING-/- THP1 cells were treated with 50μM Etoposide for 30h or 1μg/ml HT-DNA for 6h, before qRT-PCR analysis of *IL-6* mRNA. **(G)** Clonogenic survival assay of WT and IFI16-/- HaCaT cells treated with Etoposide for 24h, and grown for a further 14 days. Colonies >50 cells were counted. **(H)** Secretion of type I IFN in supernatants from WT and *IFI16-/-* HaCaT cells treated with 50μM Etoposide analysed by bioassay. **(I)** MRC-5 fibroblasts were transfected with a *IFI16*-targeting or non-targeting (NT) siRNA pool for 48h, followed by treatment with 50μM Etoposide for 6h before qRT-PCR analysis of *CCL20* mRNA. **(J, K, L)** NHEK cells were treated as cells in (I). Protein expression was analysed by immunoblotting **(J)** and *IFN-β* mRNA **(K)** or *IL-6* mRNA **(L)** expression was quantified by qRT-PCR. Data are presented as mean values of biological triplicates. Error bars indicate standard deviations. * $p<0.05$, ** $p<0.01$ Student's t-test.

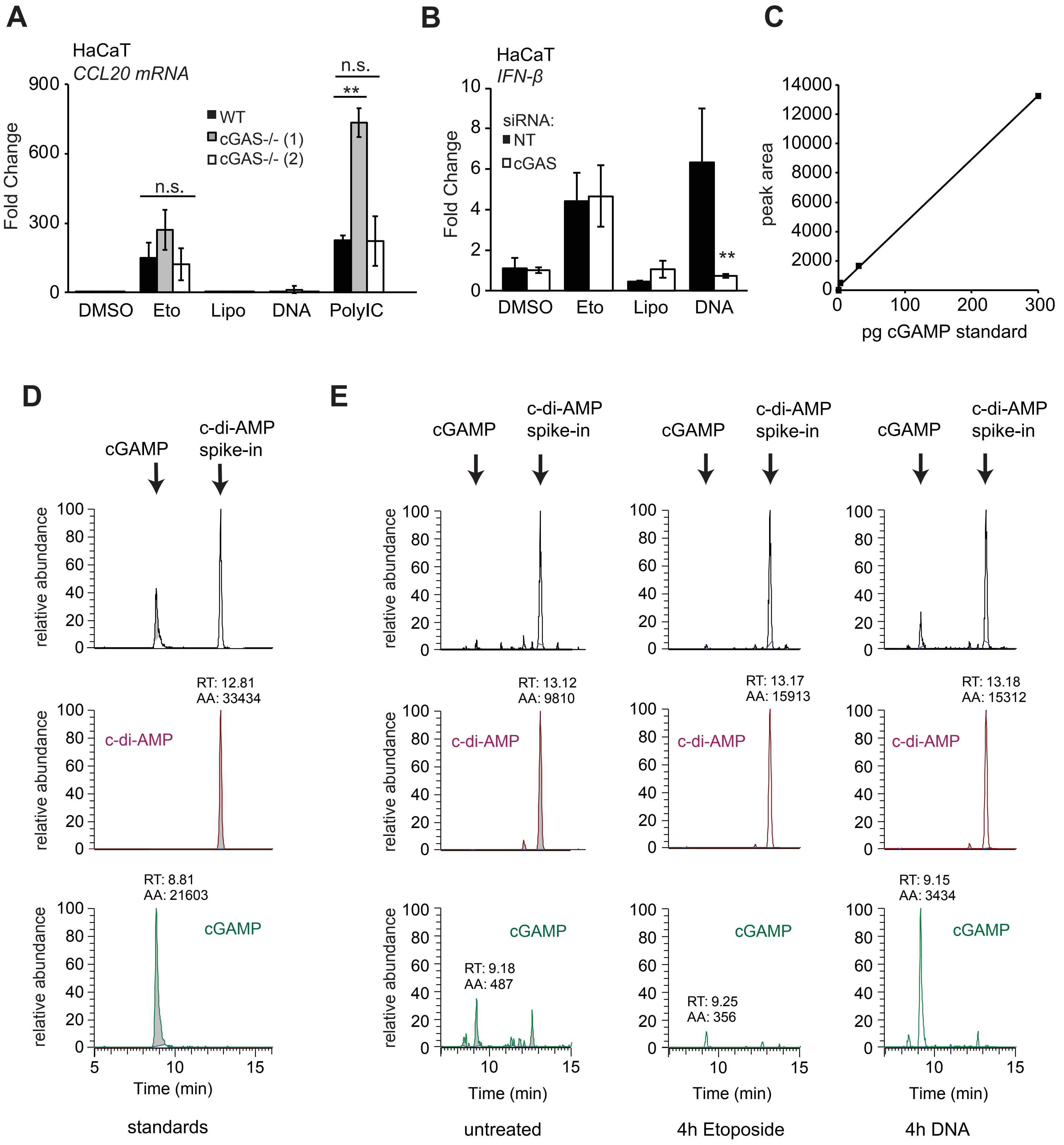

Supplementary Figure 4: cGAS and cGAMP are dispensable for the innate immune response to nuclear DNA damage. Related to Figure 4.
(A) WT and two independent *cGAS-/-* HaCaT cell clones were treated with DMSO or 50µM Etoposide, Lipofectamine, 1µg/ml HT-DNA, or 100ng/ml poly(I:C) for 6h. Expression of *CCL20* mRNA was determined by qRT-PCR. **(B)** HaCaT cells were transfected with a *cGAS*-targeting or non-targeting (NT) siRNA pool for 48h, followed by treatment with 50µM Etoposide or 1µg/ml HT-DNA for 6h before qRT-PCR analysis of *IFN-β* mRNA. **(C)** Standard curve obtained by LC-MS analysis of synthetic cGAMP standards added to untreated cell lysates prior to extraction and sample preparation. **(D)** Total and extracted ion chromatogram for cGAMP and cyclic-di-AMP standards. RT, retention time; AA peak area. **(E)** Total and extracted ion chromatograms for endogenous cGAMP and cyclic di-AMP spike-in in samples from HaCaT cells untreated, or treated with 50µM Etoposide or 1µg/ml HT-DNA for 4h. qRT-PCR data are presented as mean values of biological triplicates. Error bars are SD. ** p<0.01 by Student's t-test.

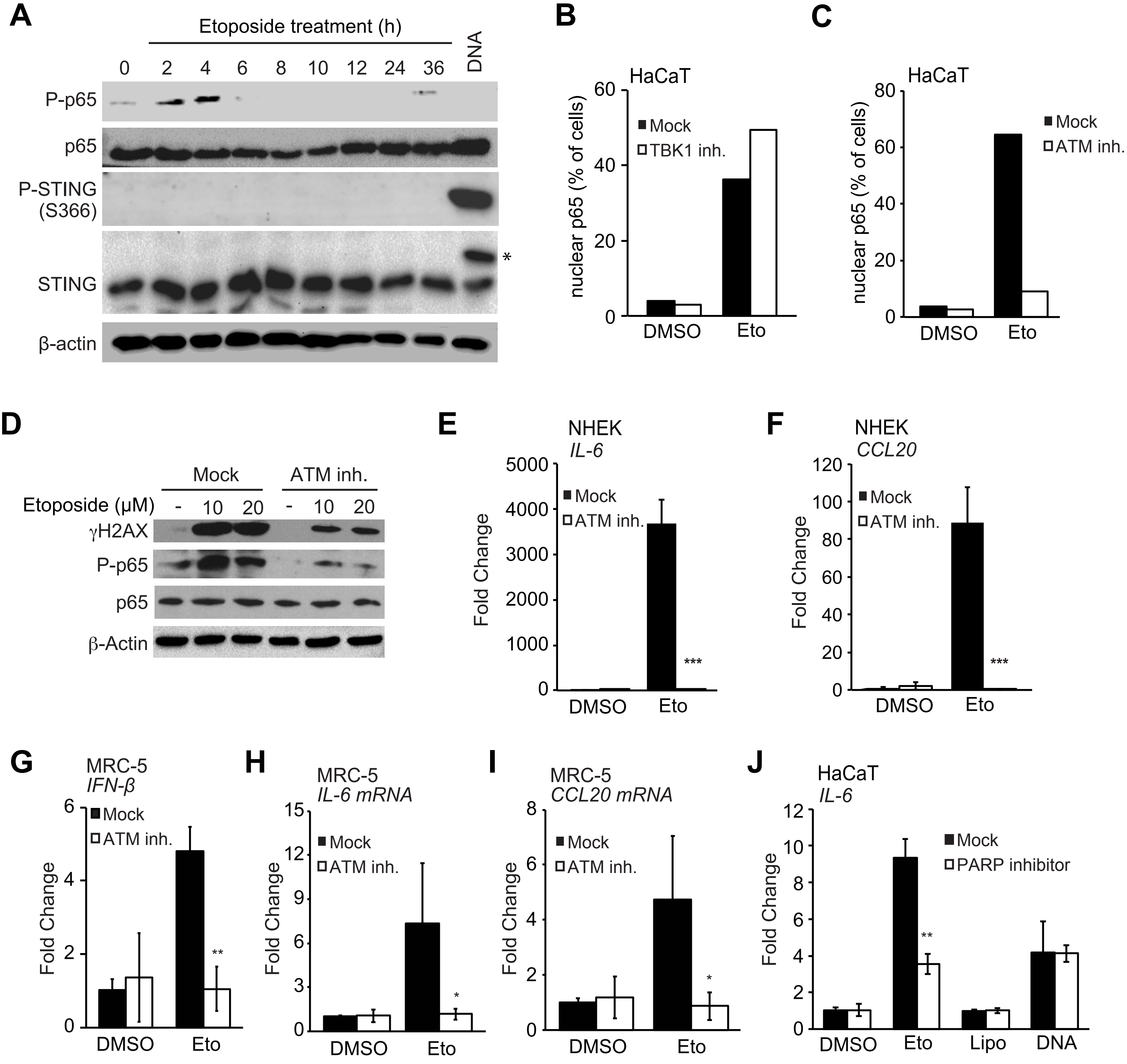

Supplementary Figure 5: Double strand breaks induce a non-canonical STING-dependent response. Related to Figure 5.

(A) HaCaT cells were treated with 50μM Etoposide for the times indicated or with 1μg/ml HT-DNA for 4h, and protein expression was analysed by immunoblotting. * indicates slower-migrating band of phosphorylated STING. **(B)** Quantification of p65 translocation as shown in **Figure 5F**. **(C)** Quantification of p65 translocation as shown in **Figure 5J**. **(D)** WT HaCaT cells were pre-treated for 1h with ATM inhibitor KU55933, or DMSO mock, before 6h stimulation with indicated Etoposide concentrations. Protein expression was analysed by immunoblotting. **(E, F)** NHEK cells were pre-treated for 1h with ATM inhibitor KU55933, or mock, before 24h stimulation with 50μM Etoposide and lysis for qRT-PCR analysis of *IL-6* **(E)** and *CCL20* **(F)** mRNA expression. **(G, H, I)** MRC-5 cells were pre-treated for 1h with ATM inhibitor KU55933, or DMSO mock, before 6h stimulation with 50μM Etoposide and lysis for qRT-PCR analysis of *IFN-β* **(G)**, *IL-6* **(H)** and *CCL20* **(I)** mRNA expression. **(J)** HaCaT cells were pre-treated for 1h with 10μM PARP inhibitor, PJ34, before treatment with DMSO, 50μM Etoposide, Lipofectamine, or 1μg/ml HT-DNA for 6h. The expression of *IL-6* mRNA was quantified by qRT-PCR. Data are presented as mean values of biological triplicates, error bars are SD. * p<0.05, ** p<0.01, *** p<0.001, Student's t-test.

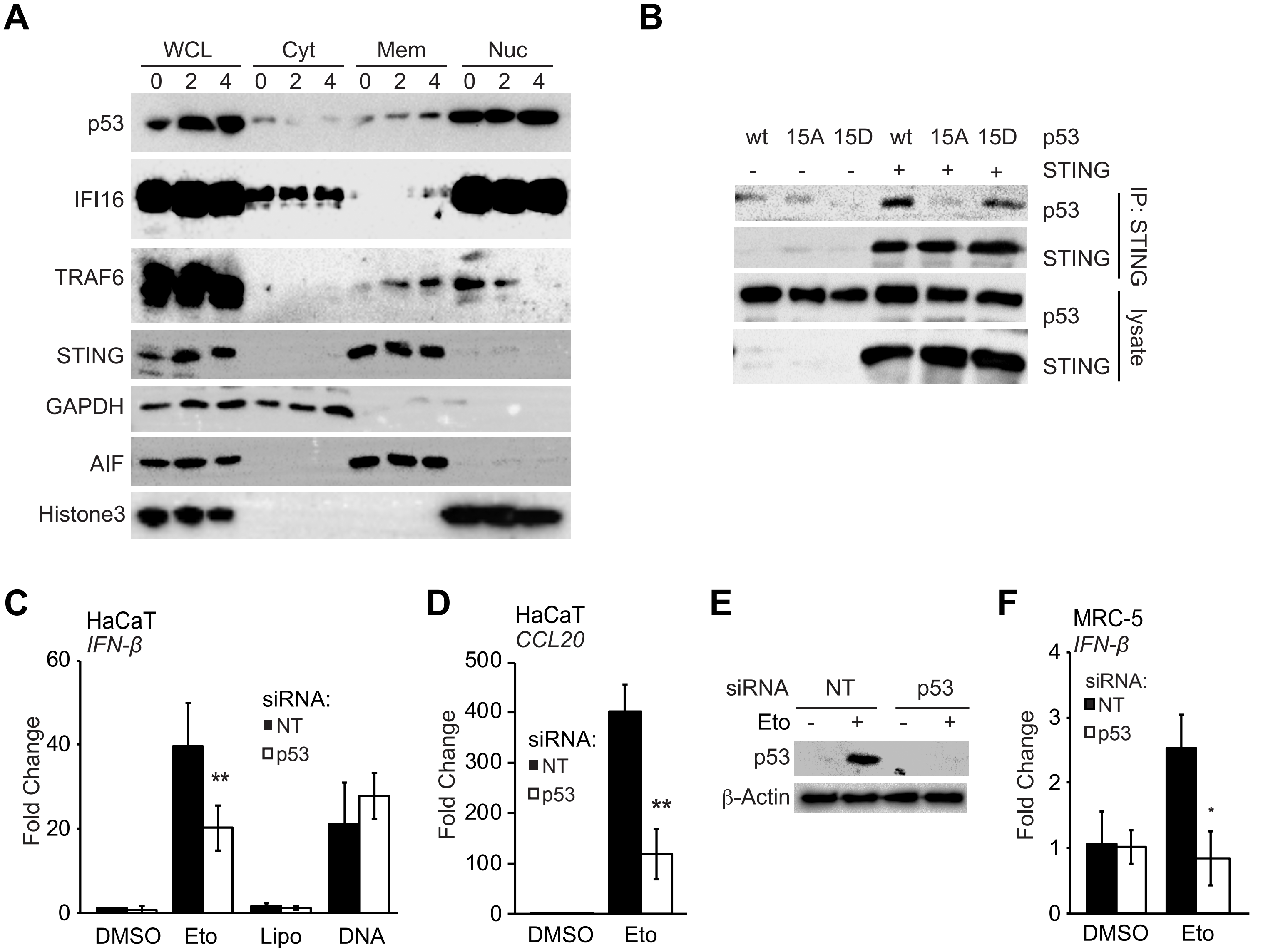

Supplementary Figure 6: The innate immune response to double strand breaks involves p53. Related to Figure 6.

(A) HaCaT cells were treated with 50μM Etoposide for indicated times before fractionation into cytoplasmic (Cyt), membrane (Mem), and nuclear (Nuc) fractions. A portion of the whole cell lysate (WCL) was retained as a control. Protein expression in fractions was analysed by immunoblotting. **(B)** HEK293T cells were transfected with expression constructs for FLAG-tagged STING and p53 plasmids expressing either wild type (WT) or S15A or S15D p53 mutants. STING was immunoprecipitated using anti-FLAG antibody. Proteins in immunoprecipitates (IP) and input lysates were analysed by immunoblotting. **(C)** HaCaT keratinocytes were transfected with non-targeting (NT) or *p53*-targeting siRNA pools for 48h, and treated with DMSO or 50μM Etoposide, mock transfected (Lipo), or transfected with 1ug/ml HT-DNA for 6h. *IFN-β* mRNA levels were analysed by qRT-PCR. **(D)** HaCaT keratinocytes transfected with siRNAs as in **(C)** were stimulated with DMSO or 50uM Etoposide and lysed after 6h. *CCL20* mRNA levels were analysed by qRT-PCR. **(E-F)** MRC-5 fibroblasts were treated as cells in (**D**). Depletion of p53 protein was assessed by immunoblotting **(E)**, and the expression levels of *IFN-β* mRNA was determined by qRT-PCR **(F)**. Data are presented as mean values of biological triplicates. Error bars indicate SD. * $p<0.05$, ** $p<0.01$, Student's t-test.

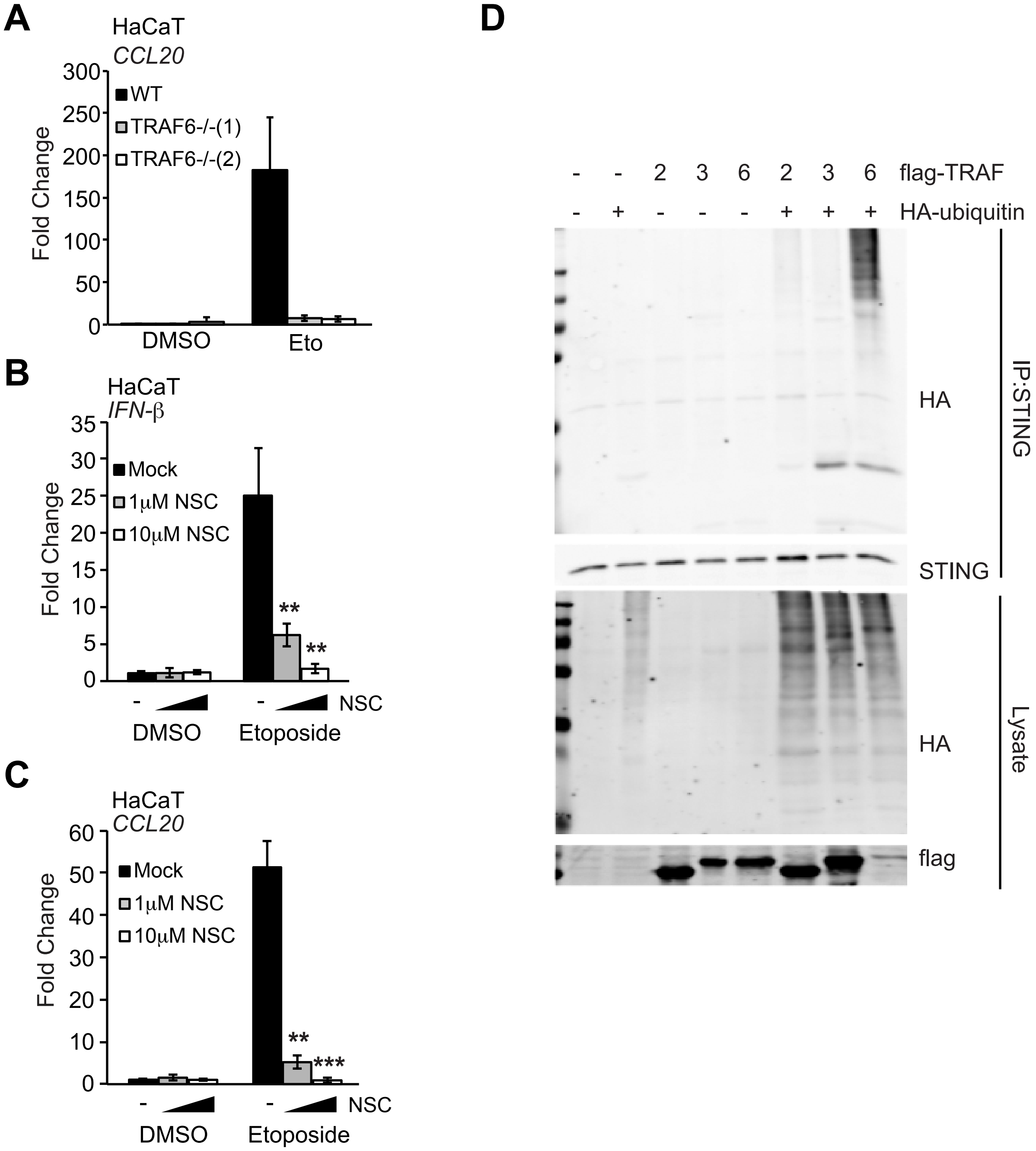

Supplementary Figure 7: The innate immune response to DNA damage involves the ubiquitylation of STING by TRAF6. Related to Figure 7.
(A) WT and two *TRAF6-/-* HaCaT cell clones were treated with DMSO or 50μM Etoposide for 6h before qRT-PCR analysis of *CCL20* mRNA. **(B-C)** WT HaCaT cells were pre-treated with indicated concentrations of Ubc13 inhibitor NSC697923, before 6h stimulation with DMSO or 50μM Etoposide and qRT-PCR analysis of *IFN-β* **(B)** and *CCL20* **(C)** mRNA. **(D)** HEK293T cells were transfected with HA-tagged ubiquitin and FLAG-tagged TRAF2, TRAF3 or TRAF6 as indicated. 24h post transfection, STING was immunoprecipitated from cell lysates, and proteins in immunoprecipitates (IP) and input lysates were analysed by immunoblotting. qRT-PCR data are presented as mean values of biological triplicates. Error bars indicate SD. ** p<0.01, *** p<0.001, Student's t-test.

Table S1: Oligonucleotide Sequences, related to STAR Methods

qRT-PCR Primer Sequences		
Primer: β-actin Forward: CGCGAGAGAAGATGACCCAGATC	This paper	N/A
Primer: β-actin Reverse: GCCAGAGGCGTACAGGGATA	This paper	N/A
Primer: IFN-β Forward: ACACTGGTCGTGTTGTTGAC	This paper	N/A
Primer: IFN-β Reverse: GGAAAGAGCTGTCGTGGAGA	This paper	N/A
Primer: IL-6 Forward: CAGCCCTGAGAAAGGAGACAT	This paper	N/A
Primer: IL-6 Reverse: GGTTCAGGTTGTTTTCTGCCA	This paper	N/A
Primer: CCL20 Forward: AACCATGTGCTGTACCAAGAGT	This paper	N/A
Primer: CCL20 Reverse: AAGTTGCTTGCTTCTGATTCGC	This paper	N/A
Primer: IRF7 Forward: CCTCTCCAGATGCCAGTCCC	This paper	N/A
Primer: IRF7 Reverse: AAGGAGCCACTCTCCGAACA	This paper	N/A
Primer: IFI16 Forward: CCGTTCATGACCAGCATAGG	This paper	N/A
Primer: IFI16 Reverse: TCAGTCTTGGTTTCAACGTGG	This paper	N/A
Primer: CXCL10 Forward: AGCAGAGGAACCTCCAGTCT	This paper	N/A
Primer: CXCL10 Reverse: AGGTACTCCTTGAATGCCACT	This paper	N/A
Primer: ISG56 Forward: CAAAGGGCAAAACGAGGCAG	This paper	N/A
Primer: ISG56 Reverse: CCCAGGCATAGTTTCCCCAG	This paper	N/A
